# A recombinant approach for stapled peptide discovery yields inhibitors of the RAD51 recombinase†

**DOI:** 10.1039/d3sc03331g

**Published:** 2023-11-21

**Authors:** Teodors Pantelejevs, Pedro Zuazua-Villar, Oliwia Koczy, Andrew J. Counsell, Stephen J. Walsh, Naomi S. Robertson, David R. Spring, Jessica A. Downs, Marko Hyvönen

**Affiliations:** a Department of Biochemistry, University of Cambridge CB2 1GA UK teodors.pantelejevs@osi.lv mh256@cam.ac.uk; b Division of Cancer Biology, The Institute of Cancer Research London SW3 6JB UK; c Yusuf Hamied Department of Chemistry, University of Cambridge CB2 1EW UK; d Latvian Institute of Organic Synthesis Aizkraukles 21 Riga LV-1006 Latvia

## Abstract

Stapling is a macrocyclisation method that connects amino acid side chains of a peptide to improve its pharmacological properties. We describe an approach for stapled peptide preparation and biochemical evaluation that combines recombinant expression of fusion constructs of target peptides and cysteine-reactive divinyl-heteroaryl chemistry as an alternative to solid-phase synthesis. We then employ this workflow to prepare and evaluate BRC-repeat-derived inhibitors of the RAD51 recombinase, showing that a diverse range of secondary structure elements in the BRC repeat can be stapled without compromising binding and function. Using X-ray crystallography, we elucidate the atomic-level features of the staple moieties. We then demonstrate that BRC-repeat-derived stapled peptides can disrupt RAD51 function in cells following ionising radiation treatment.

## Abstract 2

An approach for stapled peptide preparation in small scale using recombinant expression of peptide–protein fusions in bacteria. We use this approach to design binders of RAD51, characterise their interaction and demonstrate activity in cells.

## Graphical abstract

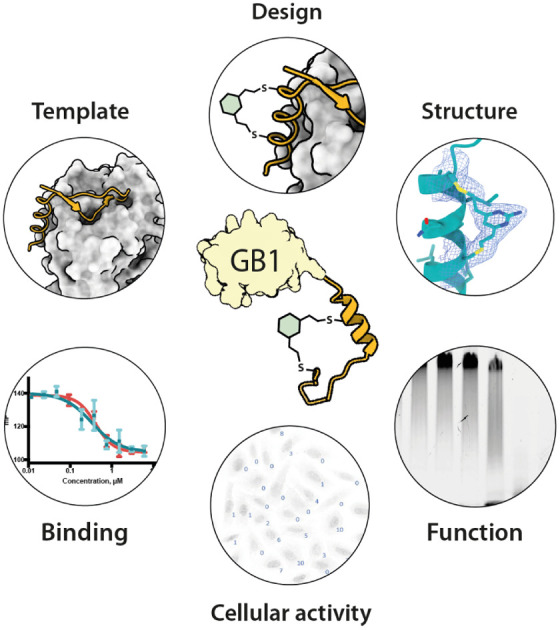

## Introduction

Peptide drugs contribute to 5% of the global pharmaceutical market and their development is hampered by a number of pharmacological pitfalls.^1^ Peptides suffer from short stability in biological fluids and low oral bioavailability, as they are prone to rapid proteolytic degradation.^2^ They are also largely unable to cross the phospholipid membrane to engage intracellular targets. Macrocyclisation aims to improve these properties by constraining the conformation of a peptide.^3,4^ This can render the peptide unable to fit into a protease active site, improving its stability and even oral bioavailability.^5,6^ Macrocyclisation can also improve a peptide's membrane permeability for intra-cellular targeting.^7^

Peptide stapling, in its broadest sense, is a macrocyclisation approach whereby the side chains of amino acid pairs within a peptide template are chemically linked to induce a more constrained conformation.^8^ In the narrowest sense, stapling is the covalent linking of α-helical peptides using ruthenium catalysed ring-closing metathesis (RCM) of non-natural amino acids bearing alkene side chains.^9,10^ More recently, alternative structural elements, such as β-hairpins and loops have been successfully utilised for stapling.^11,12^ Stapling of the nucleophilic cysteine is an attractive alternative to RCM, as it avoids the use of non-natural amino acids and metal catalysts.^13^ Moreover, stapling of cysteines can be done in mild, biocompatible conditions, allowing it to be used in the context of affinity selections of combinatorial libraries, for example, with phage or mRNA display.^14,15^

The stapling architecture, that is, the positions of the residues to be linked in the template, is a central variable in stapled peptide design. An appropriately placed staple should not interfere with binding, either by inducing an unfavourable conformation or a steric clash with the target. For helical peptides, variants are typically screened by systematically “scanning” pairs of stapled residues at fixed distances on the same side of an α-helix. For other structural motifs, in the absence of structural information, screening is more complex.

In this work, we apply a recently developed class of bis-electrophilic divinyl-heteroaryl linkers towards the recombinant production of cysteine-stapled peptides. Using the RAD51:BRC repeat interaction as a model system, we demonstrate that cysteine-stapled peptides can be prepared from small-scale bacterial cultures and screened for binding to evaluate different stapling architectures. The presented methodology provides an accessible, sustainable and rapid alternative to solid-phase synthesis and allows stapling architectures to be evaluated in three days, starting from the initial cloning experiment. We then characterise the atomic-level structural changes induced by stapling in the binding modes of these peptides, showing how both helical and non-helical structural motifs can be linked. We then demonstrate that these peptides maintain functional activity in biochemical and cellular assays.

## Results and discussion

### Small-scale recombinant preparation of stapled peptides

**Fig. 1:**
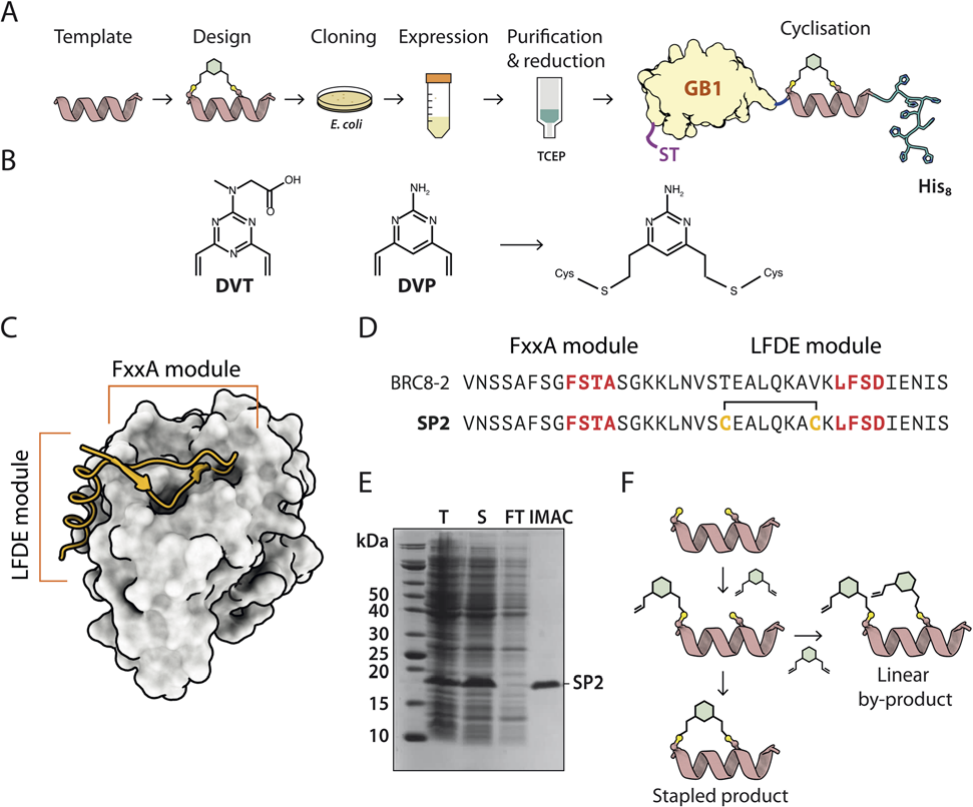
A recombinant approach for producing GB1-fused stapled peptides from small-scale *E. coli* cultures. (A) Workflow depicting the steps of stapled peptide preparation. The α-helix represents a generic peptide template. (B) Divinyl-heteroaryl linkers used in this work have been previously described.^17,18^ (C) Structural model of a BRC repeat binding to the RAD51 ATPase domain (PDB:6HQU). (D) Sequences of the linear BRC8-2 template and stapled peptide SP2. (E) Coomassie-stained SDS-PAGE of eluted GB1-SP2-His8 model peptide (F) schematic representation of the stapled peptide product and linear by-product.

Solid-phase peptide synthesis (SPPS) of a linear precursor, followed by cyclisation and HPLC purification, are typically performed to obtain stapled peptides for screening in biochemical or cellular assays.^10,16^ This involves the use of harsh chemistry, can be time-consuming, and certain peptides are not amenable to SPPS. Moreover, the process may require access to a peptide synthesiser. In order to rapidly evaluate different stapled peptide variants, we set out to develop an alternative, small-scale strategy for peptide preparation and screening, using peptides produced in bacteria. This process allows for fast, quantitative determination of *in vitro* binding affinities (Fig. 1A). Peptide design can be guided by an atomic structure of the template complexed with a target to identify mutable amino acids, but can also be performed in a structure-agnostic manner, for example, by predicting which residues are solvent-exposed and therefore not involved in binding.

To facilitate this work, we have created a bacterial expression plasmid vector, pPEPT1, where the peptide is fused to an N-terminal Strep-tag II, expression- and solubility-enhancing GB1 fusion partner (GB1) and a C-terminal octa-histidine tag (C-His_8_). The GB1 domain is relatively small (54 aa, 5.9 kDa), readily folded and expected to have minimal effect on the peptide fused to it. The C-terminal His_8_-tag is used for purification of the fusion protein, ensuring removal of possibly degraded peptide-fusions. The N-terminal Strep-tag II (8 aa, 1.1 kDa) enables tandem-affinity purification should that be needed. DNA encoding for the peptide is assembled from synthetic oligonucleotides and cloned by sequence and ligation independent cloning (SLIC) which imposes no sequence constraints to the peptide, including restriction enzyme recognition sites. The fusion protein carrying the peptide is expressed in a small scale (10 ml) *E. coli* culture, after which the cells are chemically lysed and loaded on an immobilized metal chelate affinity chromatography (IMAC) spin column. In order to prevent disulfide formation, the peptide cysteines are reduced on-resin using tris-carboxyethyl phosphine (TCEP), which does not reduce resin-bound Ni^2+^. The peptide is eluted and directly reacted with a divinyl-heteroaryl linker in a double conjugate addition step, forming the staple moiety. The cysteine-selective linkers used in this work have been reported previously both in the context of peptide stapling (divinyltriazine, DVT, Fig. 1B) and as tools for bio-conjugation of proteins (divinylpyrimidine, DVP, Fig. 1B).^17,18^

The linkers contain a six-membered heterocyclic core with two symmetrical vinyl arms that yield a single stereoisomer of a stapled product. The linkers can also be decorated with other functional moieties, such as fluorophores.^17^ After the stapling reaction, excess linker is quenched by the addition of a thiol and the reaction products are used directly in a biochemical assay. The whole process from cloning to assay can be completed in three days, it requires only a basic biochemistry laboratory set-up, and can be performed in parallel with multiple peptide designs.

RAD51 is the central recombinase enzyme that catalyses mitotic homologous recombination, a key pathway of double-strand DNA break repair.^19^ This process requires the assembly of an oligomeric RAD51 filament on resected ssDNA, mediated by an oligomerisation epitope located between its N- and C-terminal domains.^20^ RAD51 is regulated by a set of conserved ∼35 aa long BRC repeats located in the BRCA2 tumour suppressor protein.^21,22^ BRC repeats bind the C-terminal ATPase domain of RAD51 *via* two conserved tetrad motifs, FxxA and LFDE, each located on eponymous sequence modules (Fig. 1C).^21^ The FxxA motif is also found on the RAD51 oligomerisation epitope, leading to competition with the BRC repeats for the same interface. Previously, both small-molecule and linear peptide inhibitors have been developed that disrupt this interaction and inhibit RAD51 function in cells.^23,24^ We used the RAD51:BRC repeat interaction as a model system to evaluate our stapling methodology as it permits a large number of different stapling architectures to be tested in a single peptide. As template, we chose a previously identified high-affinity shuffled repeat BRC8-2, which binds a monomeric construct of RAD51 (HumRadA22) with a low-nanomolar *K*_D_ and inhibits RAD51 oligomerisation on ssDNA *in vitro*, and for which a complex structure has been determined.^25^ Model stapled peptide SP2 was designed by introducing two cysteines at the α-helical, C-terminal LFDE module of the 38-residue template (Fig. 1D). The designed cysteines replaced solvent-exposed residues *i*, *i* + 7 positions apart in a classical helical stapling fashion. Small-scale expression yielded the linear peptide fusion on a 10 nmol scale and its purity was confirmed by SDS-PAGE (Fig. 1E).

We then optimised the stapling reaction of the linear SP2 precursor. Cysteine stapling involves a two-step mechanism (Fig. 1F). The first step is a bimolecular reaction between the nucleophilic cysteine and an electrophilic arm of a linker, followed by an intramolecular ring-closing reaction of the second arm with the second cysteine. The second ring-closing step can be in competition with an undesired side-reaction, where an additional linker molecule reacts with the second cysteine. The resulting linear double-linker product is of no pharmacological utility. Typically, such side-products can be separated by HPLC, however, our method is aimed at performing initial screening without such step. To slow down the rate of second linker addition, we used pseudo-dilution of the linker *via* its step-wise addition to the linear SP2 peptide. To optimise the reaction, we examined a number of conditions, such as the rate at which the linker is added to the peptide, as well as pH and presence of TCEP. Using mass spectrometry (ESI-MS), we observed that the majority product of the initially trialled stapling reaction is the correctly linked cyclic peptide (Fig. S2 reaction a and S7†). The double linker side-product was also observed at a much lower intensity and its abundance correlated with the pace of linker addition, confirming that pseudo-dilution can aid quantitative cyclisation (Fig. S2 reactions a, b, c and S7–S9†). We assessed if TCEP can be included in the reaction to ensure cysteines remain reduced and to minimise the formation of disulfides. However, we found that at 500 μM TCEP rapidly forms undesired linear peptide-linker-TCEP adducts (Fig. S2 reactions d, e, f and S10–12†). Considering this, peptides were subsequently reduced on-resin and the stapling reaction performed immediately after elution. The final optimised reaction yielded a highly pure cyclised peptide fused to an N-terminal GB1 tag and a C-terminal His_8_-tag, as evidenced by ESI-MS (Fig. S2 reaction h and S14†).

### Affinity screening of stapled peptides

Having established a procedure for producing recombinant, cysteine-stapled peptides, we set out to prepare a variety of designs and evaluate these in a binding assay. Peptides were designed in a structure-guided manner, informed by the BRC8-2:RAD51 complex structure (PDB:6HQU).^25^ Spatial proximity and residue geometry were used as criteria for cysteine placement. Facile modelling suggests that inter-sulphur distances of 4–10 Å may be suitable for stapling with the divinyl-heteroaryl linkers. Previously it was shown that an α-helical peptide can be stapled with the DVT linker at *i*, *i* + 7 positions without perturbing its secondary structure.^18^ In a traditional α-helical stapling approach, we introduced different *i*, *i* + 7 cysteine pairs at the C-terminal LFDE module of the BRC8-2 repeat: SP1, SP8, SP9, SP15 in addition to the model peptide SP2 (Table 1). Residues selected for mutagenesis were solvent-exposed and did not form any apparent interactions with the protein. Alternatively, peptides were designed with at least one cysteine located at the β-hairpin-containing FxxA module (Table 1; SP10, SP11, SP12, SP13, SP14, SP16). Placement of cysteines in these designs was likewise guided by the structure of the complex. For example, SP12 mutates a solvent-exposed residue Phe2055 near the N-terminus of the β-hairpin and a Leu1234 at the middle of the α-helix of the LFDE module. Both side chains are solvent-exposed and located nearby (*d*_Cα–Cα_ = 11 Å), despite an 18 aa sequence distance. Some of the peptides contained complete truncations of either the FxxA or the LFDE module (SP1, SP14, SP16). We also included a negative control peptide (SP7), where the stapled residues are so far from each other that their stapling is expected to disrupt the binding mode, rendering the peptide unable to maintain the FxxA and LFDE hot-spot interactions with RAD51 simultaneously.

**Table 1:** Sequences of stapled BRC8-2 peptides and their stapling architecture, as well as the *K*_D_ values determined from the FP competition assay. *K*_D_ values were calculated from IC_50_ values using a previously reported equation. Titrations were done as single experiments with three technical replicates at each titration data point. *K*_D_ values were calculated from IC_50_ values using a previously reported equation^26^

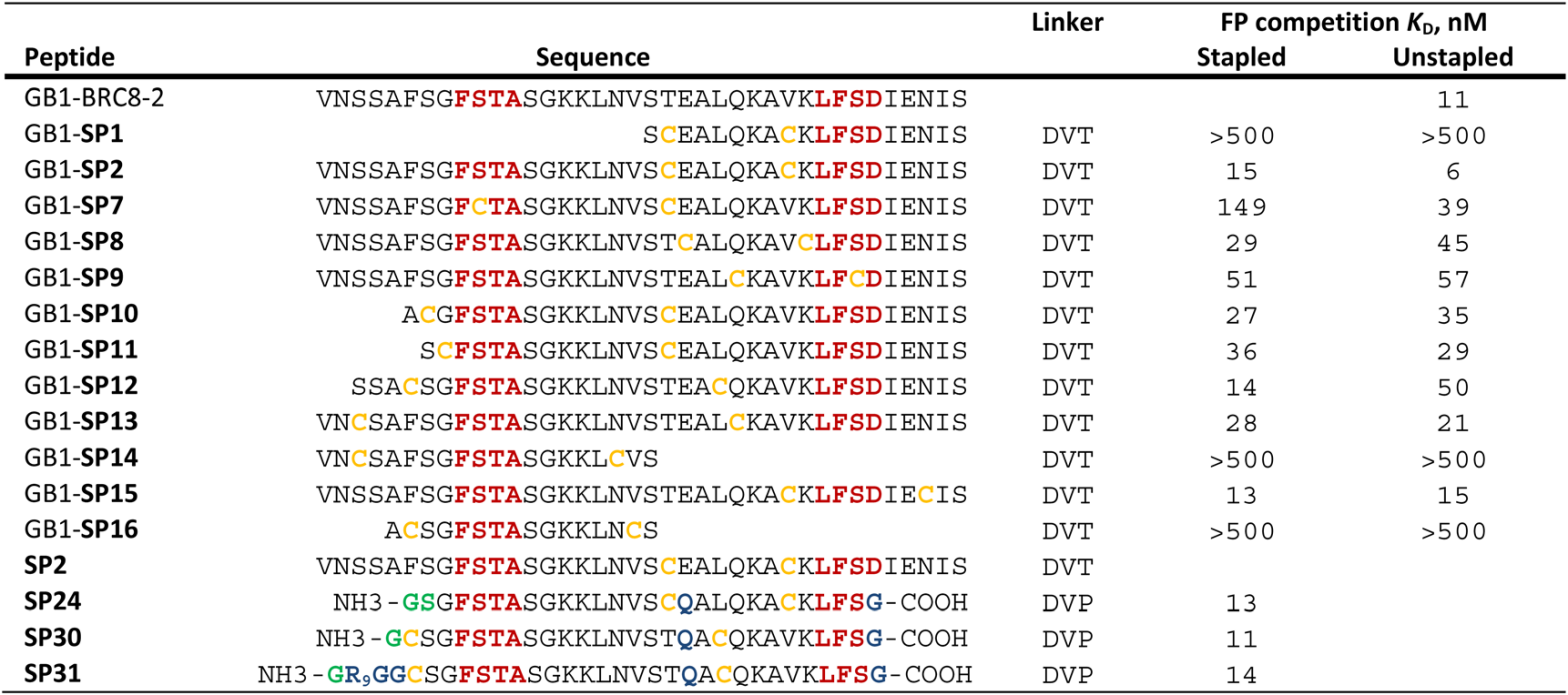

**Fig. 2:**
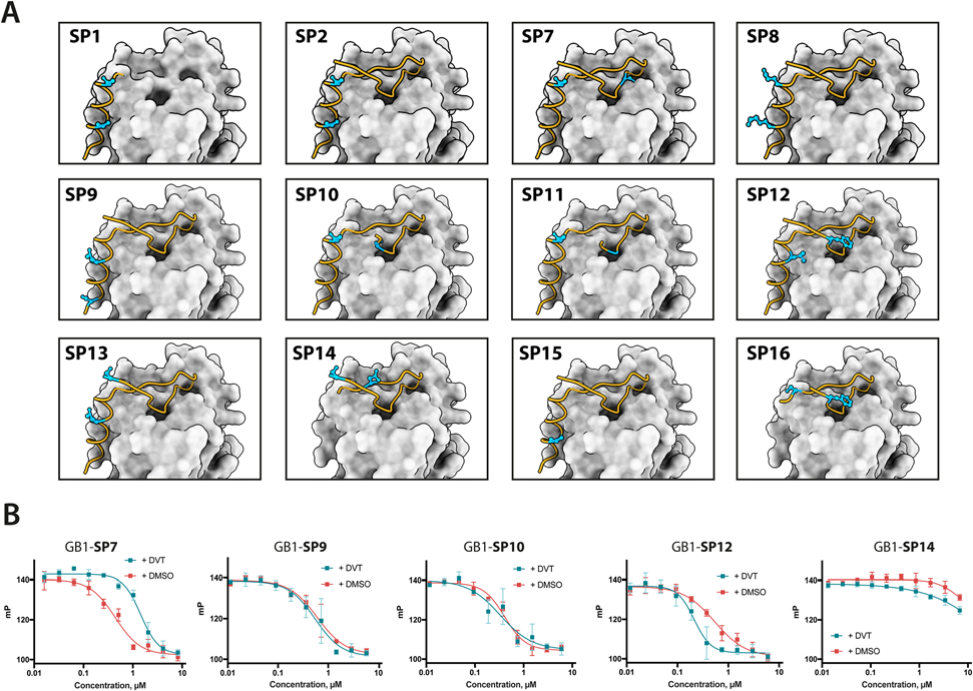
Design and screening of stapled BRC8-2 peptides. (A) Structural model of BRC8-2:HumRadA22 complex with mutable residues highlighted for selected designs. (B) Representative FP assay measurements. Red curves are unstapled, linear controls; green curves are titrations of stapled peptides. Data shown is the mean of three replicates ± SE. Data was fitted using a four-parameter logistic equation.

We prepared all stapled peptides from small-scale bacterial cultures as GB1/C-His_8_ fusions and cyclised them with the DVT linker. For peptides SP10, SP11, SP12 and SP15, correct stapling was confirmed by MS, showing a similar composition to SP2 (Fig. S15–S18†). Thus, we confirm that the pseudo-dilution approach is robust in yielding almost exclusively the cyclised product, irrespective of whether a helical or non-helical motif is being constrained. To examine the effect of introducing a covalent staple on affinity, a mock reaction was done in parallel for each peptide by splitting the IMAC elution into two halves and adding DMSO to the mock control instead of the linker. TCEP (500 μM) was included in the mock reaction to maintain the peptide in linear form by preventing disulfide formation. The peptides were tested in a fluorescence polarisation (FP) assay monitoring the displacement of a fluorescently-labelled BRC4 repeat from a monomeric version of RAD51 (HumRadA22), as reported previously.^25^ Fitted *K*_D_ values are provided in Table 1 and representative dose–response curves are shown in Fig. 2B.

The negative control peptide SP7 had a *K*_D_ of 39 nM after mock stapling, whereas the stapled product has a *K*_D_ of 149 nM, a more than a three-fold reduction in affinity, confirming the disruption of binding by an inappropriately introduced staple. All peptides containing both FxxA and LFDE hot-spot motifs bound HumRadA22 with high affinity after stapling (*K*_D_ of 15–51 nM). For these peptides, we observed minimal differences in affinity between the corresponding stapled and mock forms. Because the experiments were conducted as single titrations of technical triplicates at each concentration, we do not compare the *K*_D_ values of these high-affinity peptides. We did not observe high-affinity binding for any of the significantly truncated peptides, expanding the previous observation that both the FxxA and LFDE motifs are critical for the interaction of the BRC repeats.^23,27^ None of the repeats, either stapled or linear, bound with a higher affinity than the BRC8-2 template (*K*_D_ = 11 nM).

### Biochemical characterisation of cysteine-stapled BRC8-2 repeats

To evaluate functional and structural properties of stapled BRC repeats, we prepared some of the peptides in a tag-free form. For this, we expressed peptides in scaled-up *E. coli* cultures as fusions to an N-terminal His_8_-tag, followed by a GB1 fusion and a TEV cleavage site. Peptides were purified by IMAC, cleaved proteolytically, stapled using the pseudo-dilution approach in scaled-up reactions, and purified by HPLC, which yielded each peptide in >5 mg yield. Because both the GB1 and His-tag are N-terminal, the final products contained just the stapled peptide with a 1 to 2-residue linker at the N-terminus to ensure efficient cleavage by the TEV protease, as confirmed by LCMS (Fig. S19–S21†). Three stapled BRC8-2 repeats were produced in this manner (Table 1; SP2, SP24, SP30). SP2 and SP24 contain cysteines at identical positions in the LFDE module but differ in length and sequence. SP30 is based on SP12 and contains a distant *i*, *i* + 18 linkage across the two modules. SP24 and SP30 were significantly truncated at both termini to remove residues that are unlikely to contribute to binding, as suggested by the BRC8-2:RAD51 crystal structure. Truncation of flexible termini can aid crystallisation of a peptide:protein complex and cellular uptake. To prevent a double negative charge at the C-terminus arising from the truncation, the resulting C-terminal aspartates in SP24 and SP30 were mutated to glycine. A C-terminal glycine results in a flexible acidic tail that can mimic the Asp side chain. A different linker (DVP, Fig. 1B) was used to cyclise SP24 and SP30 instead of DVT, which was used for SP2. DVP lacks a negative charge and may benefit cellular uptake.

**Fig. 3:**
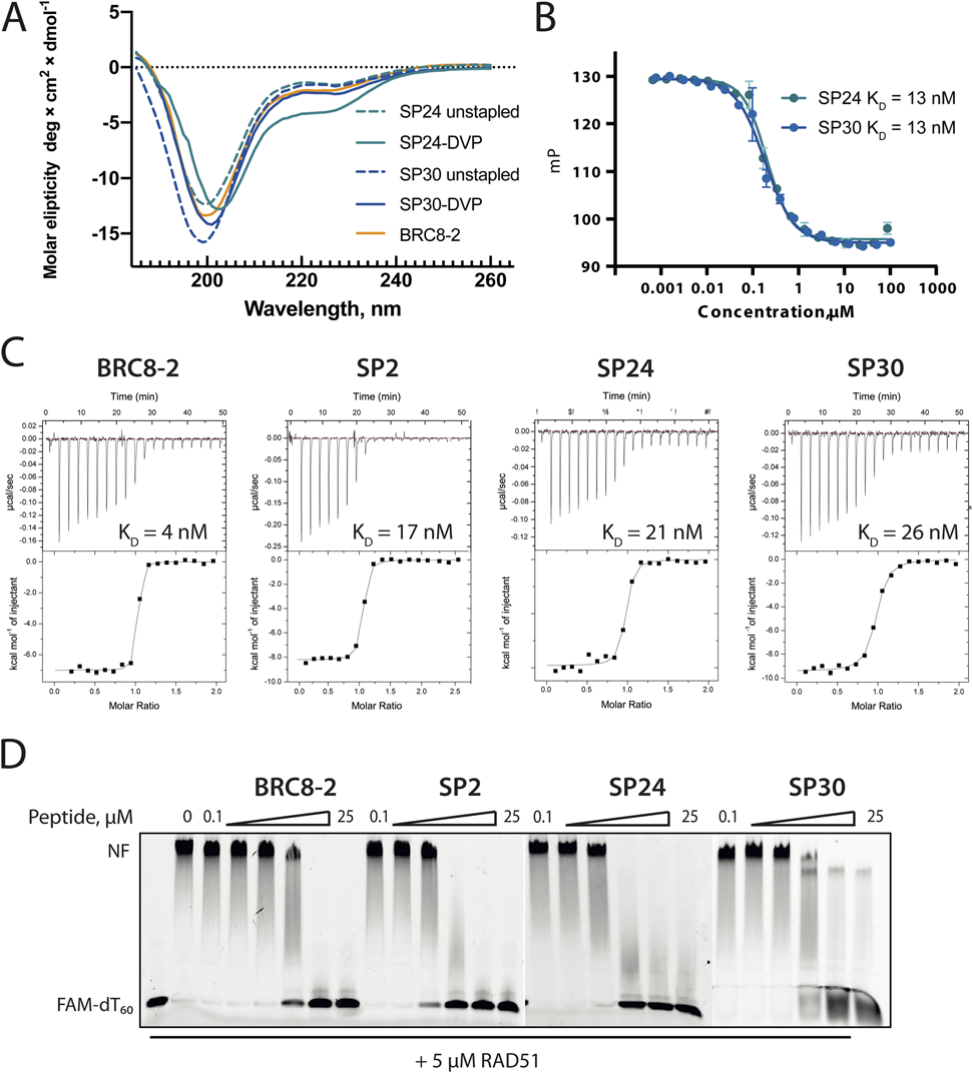
Characterisation of stapled BRC8-2 repeats purified as free peptides. (A) Circular dichroism spectra of SP24 and SP30 in stapled form and as reduced linear peptides. (B) FP competition assay measurements of SP24 and SP30 binding to HumRadA22. (C) Isothermal titration calorimetry (ITC) measurements of peptide binding to HumRadA22. (D) Electrophoretic mobility shift assay (EMSA) of RAD51:dT_60_ nucleoprotein filament incubated with varying concentrations of stapled peptides.

Previous studies have demonstrated that stapling can pre-organise unbound peptides towards the bound conformation by restricting their conformational flexibility.^18,28,29^ In particular, rationally introduced staples have been shown to enforce the secondary structures of α-helices. This effect has been hypothesised to be responsible for the improved membrane permeability of stapled peptides.^30^ We characterised the secondary structure content of the three peptides SP24 and SP30 by circular dichroism (CD), comparing both stapled and reduced linear versions of these with the BRC8-2 template (Fig. 3A). All of the tested peptides displayed a mixed secondary structure composition, with a global minimum at 200 nm corresponding to a random coil and a smaller minimum at 225 nm suggestive of an α-helix. Stapling of SP24 leads to a significant increase in α-helical character, evidenced by a more negative molar ellipticity in the 210–230 nm range, consistent with the intended stabilisation of the LFDE α-helix. No significant change in secondary structure was observed for SP30, indicating that the large cycle likely maintains a high degree of conformational flexibility.

We then evaluated the binding of free peptides to HumRadA22 using FP competition assay and isothermal titration calorimetry (Table 1 and Fig. 3B and C). In the FP assay, peptides SP24 and SP30 bound HumRadA22 with similar affinities to GB1-SP2 and GB1-SP12, which have the same respective stapling architectures, but are not truncated and carry a GB1 tag, suggesting that the initial screening approach can guide design selection. All three peptides SP2, SP24 and SP30 bound with similar low-nanomolar affinities in ITC, and the binding appears to be slightly weaker relative to the linear BRC8-2 template (Fig. 3C).

Isolated BRC repeats have been shown to disrupt the RAD51-ssDNA nucleoprotein filament by competing with the RAD51 oligomerisation interface.^31,32^ We evaluated the effect of stapled peptides SP2, SP24 and SP30 on the nucleoprotein filament using electrophoretic mobility shift assay (EMSA, Fig. 3D). A fluorescently labelled dT_60_ oligonucleotide was incubated with full-length RAD51, after which the peptides were added at different concentrations before separating the products on a polyacrylamide gel. The three peptides depolymerise RAD51 from single-stranded DNA to similar extent, suggesting that the peptides maintain function in the context of the full-length human protein.

### Structural characterisation of cysteine-stapled BRC8-2 repeats

Atomic-level characterisation of peptide:target complexes has revealed the impact of a variety of stapling chemistries on the binding modes of stapled peptides.^11,33–35^ Understanding the conformational changes induced by the staple moiety can be beneficial for the design of new binders. While it has been shown that DVT can link *i*, *i* + 7 residues on α-helical MDM2-binding peptides, the conformation of the linker and its effect on the helix geometry are unclear.

**Fig. 4:**
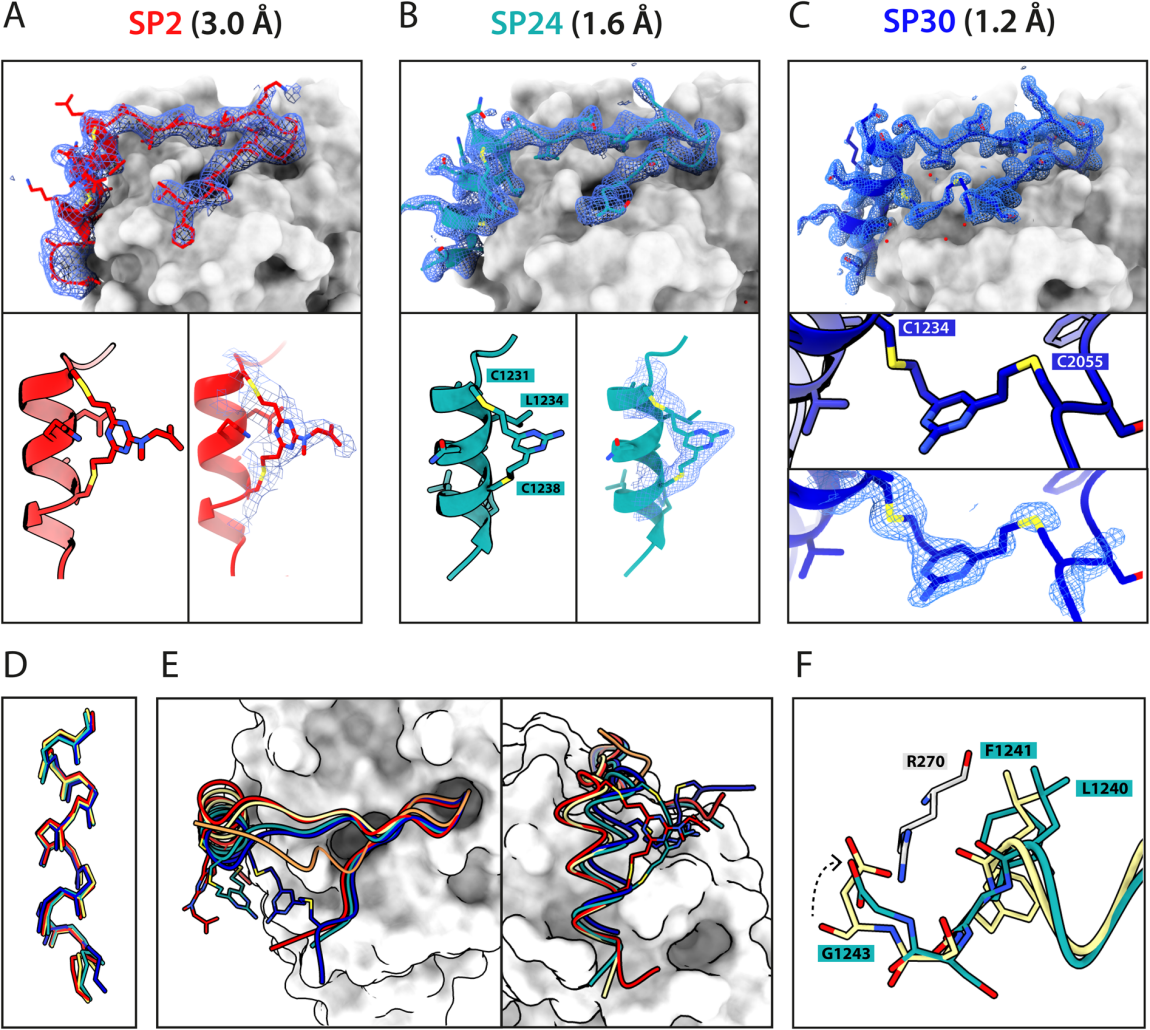
X-ray crystallographic characterisation of stapled peptide binding to HumRadA22. Blue mesh depicts weighted 2mFo-DFc electron density maps for the peptides after several rounds of refinement. Overall binding modes and zoomed-in staple moieties are shown for (A) SP2, (B) SP24 and (C) SP30. Weighted 2mFo-DFc electron density maps after modelling of the peptide are shown at 1σ level. (D) Alignment of the α-helical LFDE modules from the three stapled peptides and the BRC8-2 template complex (tan). (E) Backbone movement relative to the template structure observed for SP24 (green) and SP30 (blue). Complexes were superposed based on the HumRadA22 Cα atoms. (F) Rotation of the LFDE α-helix in SP24 results in a shift of Leu1240 and Phe1241 side chains relative to BRC8-2 template. C-Terminal Gly1243 forms a salt bridge with HumRadA22 Arg270 (HsRAD51 Arg254), mimicking an acidic sidechain.

To elucidate their binding modes, we co-crystallised SP2, SP24 and SP30 in complex with HumRadA22. Crystals of all three complexes yielded high-resolution structures, revealing the atomic-level detail of the staple moiety. In the lowest resolution SP2 complex structure, the DVT linker appears as a poorly defined “blob” in the electron density located mid-way between the two cysteine sulphurs (Fig. 4A). By contrast, in the SP24 complex structure, which connects analogous residues, the DVP linker can be observed in atomic detail and its conformation can be modelled accurately (Fig. 4B).

The two arms of the DVP linker in SP24 have acquired different orientations relative to the heterocycle core. The first arm, connected to Cys1231, has the C–C bond perpendicular to the pyrimidine ring plane, whereas in the arm linking Cys1238, the bond is nearly co-planar. In both arms, the C–C bond and the C–S bonds resemble a *trans* conformation. Thus, the staple moiety appears to experience little or no torsional and steric strain to accommodate the *i*, *i* + 7 link in an α-helical motif. The linker in SP24 does not interact with HumRadA22, but its heterocycle stacks on top of the endocyclic Leu1234 side chain, creating a small hydrophobic cluster. In both SP2 and SP24, the linker moiety lies in an unoccupied region between the side chains of endocyclic *n* + 3 and *n* + 4 residues.

The helical geometry of the C-terminal LFDE module is not perturbed either in SP2 or SP24, with near-identical distances observed between cysteine α-carbons compared to the corresponding residues in BRC8-2 (Fig. 4D). The Cα RMSD of the superimposed helices from SP2 and SP24 relative to BRC8-2 are 0.235 and 0.310 Å, respectively, indicating minimal distortion by *i*, *i* + 7 stapling. These structural observations support the application of divinyl-heteroaryl linkers as a general strategy for stapling α-helical epitopes in an *i*, *i* + 7 fashion.

In the structure of SP30 complex, the linker can be observed connecting Cys2055 and Cys1234, creating a 19 amino acid macrocycle (Fig. 4C). Electron density is clearly defined for the side chain and the linker arm on the Cys1234 side, whereas the other arm is less interpretable, possibly as a result of conformational flexibility. The pyrimidine core lies close to the surface of HumRadA22 and makes contacts with residues Gln213 and Gln217. Compared to the helical staples in SP2 and SP24, the linker arms in SP30 acquire different conformations, where both C–C bonds are perpendicular to the heterocycle core. The C–C bond at Cys1234 and C–S bond at Cys2055 are in a *gauche*-like conformation, which suggests that energetically unfavourable local strain accommodates the binding mode of the peptide.

All three stapled peptide structures have broadly similar binding modes to the parental template, with FxxA and LFDE hot-spot residues binding to their cognate hydrophobic pockets. However, the three stapled peptides do not display the extended β-hairpin observed for the BRC8-2 template. Instead, the N-terminal residues Ser2052-Gly2057 point away from the peptide and do not contribute to intra-molecular hydrogen bonding. This is anticipated in SP30, where this region is perturbed by the introduction of a staple, however, it is not clear why SP2 and SP24 display such change. It is possible that this movement of the N-terminal region is induced by crystal contacts and not by stapling. Remarkably, the LFDE modules of SP24 and SP30 undergo substantial movement relative to HumRadA22 and the rest of the peptide (Fig. 4E). The observed motion affects more than half of the peptide, encompassing residues Lys1226 to Glu1243. The movement is concomitant with a rotation of the LFDE α-helix around its helical axis, leading to the re-organisation of Leu1240 and Phe1241 side chains in their cognate hydrophobic clefts (Fig. 4F). It is reasonable to hypothesise that the shift of the LFDE module is a consequence of the above-described C-terminal Asp1243Gly mutation, which was introduced to reduce the overall negative charge of the peptide. The carboxylate of the terminal glycine residue is one carbon shorter compared to an aspartate side chain, and rotation of the LFDE helix brings it closer to HumRadA22 Arg270 (HsRAD51 Arg254) in the SP24 structure, to maintain the salt bridge. Interestingly, despite this large-scale shift, the peptides maintain low nanomolar binding affinities, suggesting that the RAD51:BRC interface can accommodate structural plasticity not observed in previous studies.

### Cysteine-stapled BRC repeat inhibits RAD51 foci formation in cells

Following treatment of cells with ionising radiation (IR), RAD51 translocates to the nucleus and forms foci at sites of DNA damage that are visible by immunofluorescence (IF) microscopy. Previously, we have shown that transient expression of a GFP-BRC8-2 peptide fusion leads to the efficient disruption of IR-induced RAD51 foci in the U2OS osteosarcoma cell line.^25^ With some exceptions, the large size of peptides is detrimental to membrane permeability and restricts their use to extra-cellular targets. To circumvent this, various cationic cell-penetrating motifs have been conjugated to peptides and other biomolecules to aid cellular uptake. Arginine-rich cell-penetrating peptides (CPPs) are one of the most commonly applied motif that has been shown to internalise by inducing membrane multilamerality and forming of a fusion pore.^36^ A prior study reported the successful application of nona-arginine (Arg_9_) motif to aid the cellular uptake of a BRC4-derived linear peptide to disrupt RAD51 function in cells.^24^ To enhance cellular uptake of our stapled BRC repeats, we prepared a similar derivative of SP30, called SP31, by recombinantly introducing an N-terminal Arg_9_ sequence (Table 1). We confirmed that SP31 has high affinity towards HumRadA22 using both FP and ITC (Fig. S3†).

**Fig. 5:**
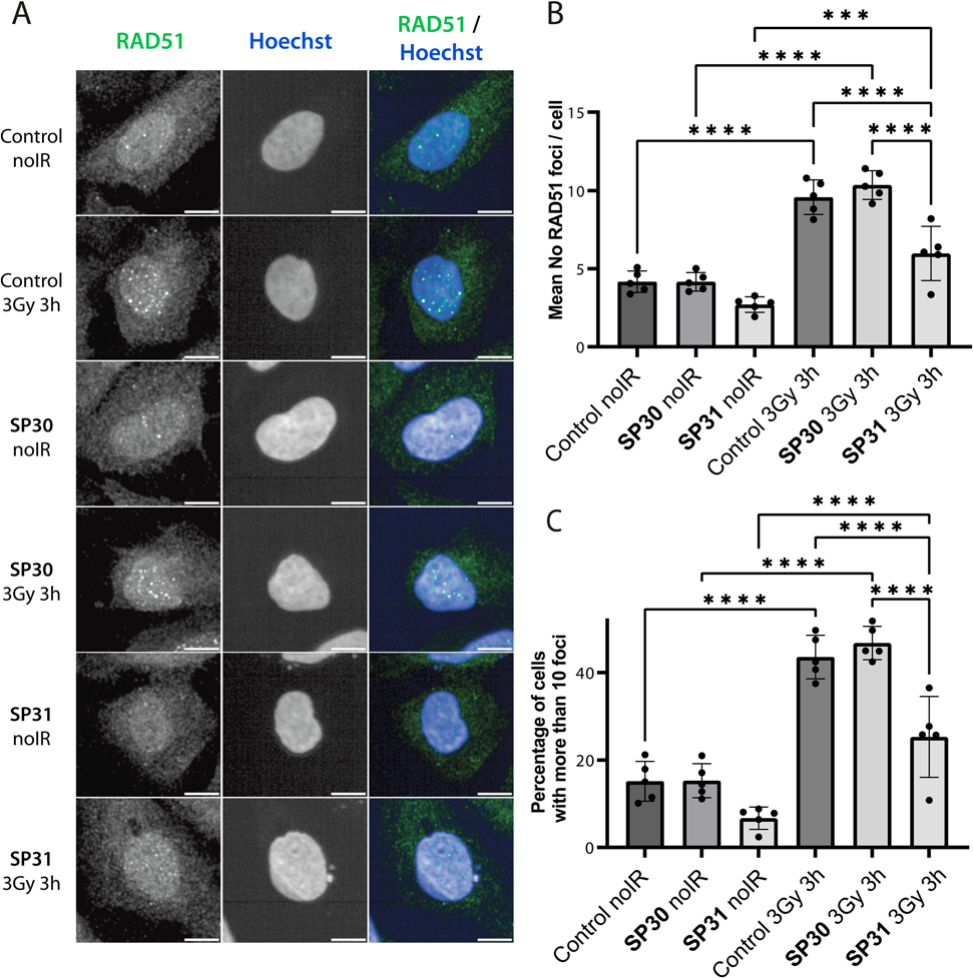
Stapled peptide SP31 disrupts RAD51 foci formation in human U2OS cells. (A) Representative immunofluorescence images of U2OS cells incubated with SP30 (40 μM, SP30), SP31 (40 μM, SP31) or vehicle alone (control) for 1 hour, after which they were treated with 3 Gy ionising radiation (3 Gy 3 h) or no radiation (noIR) and allowed to recover for 3 hours. Cells were stained with α-RAD51 and Hoechst 33342 as indicated. Scale bar is 10 μm. (B) Bar graph showing the average of the mean counts of RAD51 foci per cell from five independent biological experiments. Data are presented as mean ± SD, *** – *P* < 0.001, **** – *P* < 0.0001 using ANOVA test followed by Tukey's method. (C) Bar graph showing the average percentage of U2OS cells with more than 10 RAD51 foci per cell. Data are presented as mean ± SD, **** – *P* < 0.0001, using ANOVA test followed by Tukey's method.

We monitored the ability of SP30 and SP31 to disrupt IR-induced RAD51 foci formation using IF. Cells were pre-incubated with the peptides or with vehicle (control), after which they were either treated with no radiation or with 3 Gy IR and imaged after a 3 hours recovery. We observed RAD51 foci in the absence of peptide and IR treatment, which likely reflects recombination events associated with the basal replicative stress of U2OS cells (Fig. 5 and S4†). As expected, IR treatment of control cells leads to an apparent increase in the number of RAD51 foci (Fig. 5 and S4†). Notably, addition of SP31, but not SP30, significantly reduces the number of RAD51 foci in both IR-treated and untreated cells (Fig. 5 and S4†). The IF data demonstrates that the Arg_9_-fused peptide SP31 is able to pass cell membrane and sequester RAD51, preventing its oligomerisation on DNA and foci formation. We also tested whether a linear version of SP31 (L31) also affects IR-induced and baseline foci counts in five independent experiments (Fig. S5 and S6†). Surprisingly, L31 was able to inhibit RAD51 foci formation in some of the experiments, similarly to SP31, whereas in others it had little to no effect relative to control, resulting in a very large variation in quantitation of these experiments. These observations suggest that L31 may be more susceptible to proteolytic degradation, but more in-depth analytical studies would be necessary to ascertain this. It is possible that in some of the experiments the unstapled cysteines of L31 formed a disulfide bridge in the oxidising environment of cell culture, which protected it against proteolytic degradation, similarly to chemical stapling.

## Conclusion

Finding an optimal stapling architecture requires the testing of many peptide sequences. Preparation of peptide macrocycles, including stapled peptides, is traditionally performed using specialist equipment such as peptide synthesisers, lyophilisers and HPLCs and uses toxic solvents such as dimethylformamide (DMF) and *N*-methyl-2-pyrrolidone (NMP). This renders solid-phase peptide synthesis challenging from a sustainability standpoint, calling for specific waste-disposal procedures. Commercial procurement of a large number of synthetic peptides can be cost-prohibitive in academic research, with typical prices at $10 USD per amino acid for 90% pure product. In this work we have presented a methodological workflow that allows stapled peptides to be prepared in sufficient amounts for quantitative evaluation of their binding using standard biochemistry equipment. Utilising parallel, small-scale bacterial expression of peptides as small fusion proteins with tags for rapid purification in combination with cysteine-reactive linkers, we demonstrated that stapled peptides can be prepared in three days from the initial cloning steps. The insert encoding for the peptide is generated with oligonucleotides that are readily accessible and inexpensive, compared to solid-phase peptide synthesis; a pair of oligos needed for a 30 residue peptide costs *ca.* £20 GBP. The GB1 fusion only improves expression levels but also increases peptide solubility and allows for quantification by UV absorbance measurements at 280 nm. We anticipate that this method can be expanded in throughput by performing purification and stapling steps in 96-well format. This approach will be particularly suited for proof-of-concept studies with the aim of modulating protein–protein interactions. However, compared to use of purified synthetic peptides, some limitations warrant discussion as well. In some contexts, the GB1 fusion may exert a detrimental effect on binding due to steric repulsion with the target protein and we advise to introduce a longer flexible amino acid linker to avoid this. Other small fusion partners could also be used, provided they do not contain cysteines, and our pPEPT1 vector is built so that the GB1 can be replaced easily with, for example, SUMO (small ubiquitin-like modifier) protein. Secondly, the method involves a single affinity purification step with the C-terminal His-tag, therefore proteolysis products may be co-purified with the full-length fusion and interfere with downstream assays. To counter this, we have included in the plasmid an N-terminal Strep-tag II for tandem affinity purification. Thirdly, the presented method does not allow for facile incorporation of non-natural amino acids, as is possible with solid-phase synthesis, hence the accessible chemical space is more limited.

The small-scale preparation of GB1-fused, stapled BRC8-2 peptides yielded sufficient product for testing in an FP competition assay. We envisage that peptides prepared in this manner can be used in a range of biophysical and biochemical assays, such as surface plasmon resonance (SPR), homogeneous time-resolved fluorescence (HTRF), dynamic scanning fluorimetry (DSF) and others. The methodology is not suitable for cell assays where the target is intracellular, as the bulky GB1 tag is most certainly preventing cellular uptake. Instead, it is aimed at cases where a purified target is available for *in vitro* measurements. We anticipate that the described method can be used with alternative cysteine-reactive bis-electrophilic linkers, such as bis-haloacetamides, however, reaction selectivity will have to be optimised and it is not certain that similar levels of correctly stapled product can be obtained.

Structure-guided design is a powerful tool in drug-discovery. Here we have used the previously published BRC8-2:RAD51 complex structure to inform the design of a structurally diverse set of stapled peptide variants. Using our recombinant workflow, we have prepared and evaluated these designs, showing that different structural motifs in the BRC repeat can be stapled without impairing affinity. We also show that BRC repeats can be significantly truncated with no disruption to binding. However, both FxxA and LFDE motifs are indispensable, supporting previous studies. Our crystallographic analysis reveals the different structural features of the staple moiety, which will be beneficial for future studies utilising these linkers. Finally, we show that these peptides potently de-polymerise RAD51 from ssDNA *in vitro* and, when fused to a cell-penetrating peptide, inhibit RAD51 foci formation in cells. The stapled peptides described in this work represent a novel pharmacological modality for targeting RAD51 function by competing with its self-oligomerisation and interaction with BRCA2.

## Data availability

Crystallographic data for stapled peptide complexes with HumRadA22 has been deposited at the PDB under accession numbers 8C3J (SP2), 8BR9 (SP24), 8C3N (SP30).

## Author contributions

Conceptualization, T. P., P. Z.-V., J. A. D., M. H.; methodology, T. P., P. Z.-V., J. A. D., M. H.; formal analysis, T. P. and P. Z.-V.; investigation, T. P., P. Z.-V., O. K.; resources, T. P., A. J. C., S. J. W., N. S. R., D. R. S., J. A. D., M. H.; data curation, T. P., P. Z.-V.; writing: original draft, T. P., M. H.; writing: reviewing and editing, T. P., P. Z.-V., A. J. C., S. J. W., D. R. S., J. A. D., M. H.; visualization, T. P., P. Z.-V.; supervision, D. R. S., J. A. D., M. H.; project administration, T. P. and M. H.

## Conflicts of interest

There are no conflicts to declare.

## Supplementary Material

SC-014-D3SC03331G-s001
