# Supplementary material for: A recombinant approach for stapled peptide discovery yields inhibitors of the RAD51 recombinase
Source: Chem Sci. 2023 Nov 21;14(47):13915–23. doi: 10.1039/d3sc03331g (PMC10699557; doi:10.1039/d3sc03331g)
Supplement: SC-014-D3SC03331G-s001 [file SC-014-D3SC03331G-s001.pdf]

Electronic Supplementary Material (ESI) for Chemical Science.
This journal is © The Royal Society of Chemistry 2023

Supporting information

A Recombinant Approach For Stapled Peptide Discovery Yields Inhibitors of the RAD51 Recombinase

Teodors Pantelejevs[1,4*], Pedro Zuazua-Villar[2], Oliwia Koczy[1], Stephen Walsh[3], Naomi Robertson[3], Andrew Counsell[3], David Spring[3], Jessica Downs[2], Marko Hyvonen[1*]

[1] Department of Biochemistry, University of Cambridge, CB2 1GA, UK

[2] Division of Cancer Biology, The Institute of Cancer Research, London SW3 6JB, UK

[3] Yusuf Hamied Department of Chemistry, University of Cambridge, CB2 1EW, UK

[4] Latvian Institute of Organic Synthesis, Aizkraukles 21, Riga, LV-1006, Latvia

Keywords: stapled peptides, BRC repeat, BRCA2, RAD51

Supporting information 1

A Recombinant Approach For Stapled Peptide Discovery Yields Inhibitors of the RAD51 Recombinase 1

Materials and methods 3

Materials 3

Small-scale preparation of GB1-fused stapled peptides 5

Preparation of stapled peptides in un-tagged form 6

Purification of HumRadA22 7

Purification of full-length human RAD51 7

Fluorescence polarisation competition assay 8

Circular dichroism spectroscopy 9

Electrophoretic mobility shift assay (EMSA) 9

X-ray crystallography 10

Cell line 10

Immunofluorescence 10

X-ray crystallography statistics 12

Oligonucleotides used for cloning of peptides 13

pPEPT1 vector 14

Optimisation of the small-scale stapling reaction 15

SP31 *in vitro* binding data to HumRadA22 15

Rad51 foci inhibition data for stapled peptides SP30 and SP31 16

Rad51 foci inhibition data for linear peptide L31 17

Mass spectra 19

Mass spectra of reaction optimisation conditions 19

Mass spectra of representative GB1-fused stapled peptides 23

Mass spectra of free stapled peptides 25

HPLC analysis of free peptides 26

Materials and methods

Materials

Reagent	Source	Identifier
**Cell lines**		
U2OS	ATCC	HTB-96
**Antibodies**		
Rabbit anti-RAD51	Santa Cruz biotechnology	sc-8349
Mouse anti-rabbit Alexa Fluor 488	Life Technologies	A21206
**Bacterial strains**		
E. coli T7 Express chemicompetent cells	Prepared in-house from a New England Biolabs glycerol stock	C2566H
E. coli BL21 (DE3) Rosetta	Prepared in-house from a Novagen stock	70954-3
**Recombinant DNA and oligonucleotides**		
pPEPT1 plasmid	This work. See **Figure S1.**	Adgene #195001
pOP3BT	Hyvonen Lab, University of Cambridge	Addgene #112603
pEXP-NHis-GB1	Hyvonen Lab, University of Cambridge	Addgene #112565
Oligonucleoides for cloning peptides.	Synthesised by Sigma-Aldrich. See **Table S2**.	
**Purified proteins and peptides**		
GB1-peptide fusions	This work	
Un-tagged BRC repeat peptides	This work	
Fluorescein-BRC4	Gift from Dr Laurens Lindenburg, previously reported in [1]	
Human RAD51	This work	
HumRadA22 protein	This work, previously described in [2]	
Lysozyme	Sigma-Aldrich	1.05281
T4 DNA polymerase	New England Biolabs	M0203S
DNAse I	Sigma-Aldrich	DN25

TEV protease	Prepared in-house using bacterial expression from the pRK793 plasmid (Addgene #8827)	
Phusion DNA polymerase	New England Biolabs	M0530S
*Bsa*I	New England Biolabs	R3733S
*Hind*III	New England Biolabs	R0104S
*Xho*I	New England Biolabs	R0146S
*BamH*I	New England Biolabs	R3136S
**Chemicals**		
DVP linker	Spring lab, University of Cambridge, described previously[3]	
DVT linker	Spring lab, University of Cambridge, described previously[4]	
TCEP	Melford	T26500
IPTG	Melford	I56000
Ampicillin	Melford	A40040
AEBSF	Melford	A20010
Ni-NTA agarose	Cube Biotech	31103
NEBuffer 2.1	New England Biolabs	B6002S
2xYT medium	Formedium	AIM2YT0210
Penicillin/streptomycin	Sigma-Aldrich	P4333
Bovine serum albumin	Sigma-Aldrich	A9647
Fetal bovine serum	Sigma-Aldrich	F7524
DMEM – high glucose	Sigma-Aldrich	D6429
Triton X-100	Sigma-Aldrich	T8787
ProLong Gold Antifade Mountant with DAPI	ThermoFisher	P36935
**Columns and consumables**		
HiTrap Heparin 5 ml	Cytiva	17040703
PD-10 desalting column	Cytiva	17085101
384-well flat-bottom microplates	Corning	3821

Small-scale preparation of GB1-fused stapled peptides

Peptide-coding DNA was designed using the DNAWorks online application,[5] optimising codon usage for *E. coli* expression. DNA oligonucleotides for the assembly of these fragments were generated using the same software and 15-20 nt linkers were appended to the 5' ends of the outermost forward and reverse oligos for sequence and ligation independent cloning (SLIC). Oligonucleotides used for assembly of the fragments are shown in **Table S2**. The inserts were then synthesised by assembly PCR using Phusion DNA polymerase (New England Biolabs) with standard reaction conditions. Each assembly PCR reaction contained 1 μM of the outermost oligos and 0.02 μM of each internal oligo. Inserts were then purified by gel extraction using the GeneJet gel extraction kit (ThermoScientific). Sequence and ligation-independent cloning (SLIC) was used to clone the inserts into the pPEPT1 vector (Addgene #195001, **Figure S1**) digested with the *Bsa*I restriction enzyme. Using *BsaI* allows the peptide to be cloned in a seamless fashion as cleaves outside of its recognition site. 10 μl SLIC reactions contained purified insert at 2-10 ng/μl and digested vector at 4-20 ng/μl in 1x NEB Buffer 2.1 (New England Biolabs). 0.6 U of T4 DNA polymerase (New England Biolabs, M0203S) was added to each reaction and incubated for 1-5 min at RT, after which either dGTP or dCTP was added to a final concentration of 10 mM to stop exonuclease activity. Reaction was further incubated for 1 minute at RT, after which it was heated for 5 minutes at 65 °C in a PCR cycler to deactivate the T4 polymerase, after which the PCR tubes were left at room temperature for 10-20 minutes for the complementary resected ends of the insert and vector to anneal. Reaction mixtures were then used to transform 50 μl of chemicompetent T7Express *E. coli* cells by heat shock and transformants were plated on LB agar plates supplemented with ampicillin (100 μg/ml). Individual colonies were transferred to a replica plate and presence of insert was determined by colony PCR using the forward primer used for the assembly PCR and T7 terminator primer (GCTAGTTATTGCTCAGCGG). For expression, clones carrying an insert were used to inoculate 10 ml 2xYT bacterial cultures supplemented with ampicillin (100 μg/ml). The cultures were grown in a 50 ml centrifuge tube at 37 °C overnight with the tube lid slightly unscrewed and fixed with tape to ensure aeriation. Next day, protein expression was induced by the addition of IPTG (400 μM) for three hours at 37°C, after which cells were harvested by centrifugation. Cell pellets were then either frozen for future use or used directly for purification of peptides. Correct inserts were confirmed later by Sanger sequencing with the T7 terminator primer.

Cells were resuspended in 1 ml of lysis buffer: PBS, 20 mM imidazole, 1 mM TCEP, 1 mM EDTA, 0.1% Triton X-100, 0.2 mg/ml lysozyme, 1 mM AEBSF, 10 µg/ml DNase I. Lysate was incubated for 10 min at room temperature on a rotating mixer. Lysates were spun down in a 2 ml tube on a bench-top centrifuge at 15 000 g for 10 min and supernatant collected by aspiration. 200 µl of 50% (v/v) slurry of Ni-NTA agarose resin (Cube Biotech, !31103) was washed twice with 1 ml of MilliQ water and resuspended in 200 µl PBS. The resin was mixed with the soluble lysate and incubated on a rotating mixer for 5 min at room temperature, after which it was applied in two portions to a 0.5 ml micro-spin chromatography column and centrifugated for 1 min at 1000 x*g* to remove flow-through. Same centrifuge settings were also used for subsequent wash and elution steps. The resin was washed with a total of 1 ml of PBS + 20 mM imidazole containing 1 mM TCEP, followed by a 0.5 ml wash using the same buffer without any reducing agent. The second wash step is essential for the removal of any residual TCEP that can form undesired side-products upon reaction with the divinyl-heteroaryl linker. The GB1-BRC repeat was eluted with 0.5 ml PBS + 200 mM imidazole, and the elution immediately used for subsequent stapling reactions.

The eluted sample was split into two 250 µl parts. 2 mM DVT or DVP linker solution in DMSO was gradually titrated into the stapling reaction to achieve pseudo-dilution conditions. Different linker titration schemes were initially trialled and are shown in **Figure S2**. Most optimal linker titration was observed for reaction **h** (**Figure S2**) and was used for subsequent preps. At the same time, an identical volume of DMSO control without any linker was added to the other 250 µl peptide solution. 1 mM TCEP was added to the control reaction but not the stapling reaction to maintain free sulfhydryl groups in the control peptides. At the end of the titration, reactions were quenched with 2 mM DTT.

Preparation of stapled peptides in un-tagged form

Peptides were cloned in an identical fashion to the GB1-peptide-His_8 constructs, except different expression vectors, pOP3BT and pEXP-GB1, were used (Addgene #112603 and #112565, respectively). The vectors contain an N-terminal instead of a C-terminal His-tag. T7Express *E. coli* cells carrying the plasmids expressing GB1-fused BRC repeat were plated directly from glycerol stocks of sequence-verified clones onto LB agar supplemented with ampicillin (100 µg/ml) and grown overnight at 37 °C. Next day, cells were scraped to inoculate separate flasks containing 1 L of 2x YT medium supplemented with 100 µg/mL ampicillin. Cultures were grown at 37 °C until OD_{600} of ~1, after which expression was induced with 400 µM IPTG for 3 h. Cells were resuspended in 25 mL of IMAC buffer A (50 mM Tris-HCl pH=8.0, 150 mM NaCl, 20 mM imidazole) and frozen. Later, cells were thawed and

supplemented with DNase I (100 µL, 2 mg/mL) and AEBSF (1 mM), and lysed on an Emulsiflex C5 homogenizer (Avestin) or by sonication. Cell lysate was centrifuged at 40000 x*g* for 30 min and supernatant collected. GB1-BRC lysate was loaded on a 3 mL Ni-NTA agarose matrix (Cube Biotech), after which column matrix was washed with 10 column volumes Nickel Buffer A. GB1-BRC repeat was eluted with 12 ml nickel buffer B (50 mM Tris-HCl pH 8.0, 150 mM NaCl, 200 mM imidazole). The eluent was buffer exchanged back into nickel buffer A on a PD-10 desalting column (Cytiva). Buffer exchanged GB1-BRC fusion (~18 ml) was incubated with 100 µL of 2 mg/ml TEV protease overnight at 4°C. The GB1 tag was then removed from the solution by a second Ni-NTA affinity step, collecting the flow-through that contains the BRC peptide. The flow-through was acidified with HCl to pH 2-4 and acetonitrile was added to 10%, after which the solution was centrifuged at 10000 x*g* for 15 min and supernatant collected. The acidified flow-through was then applied to an ACE C8 300 4.6 x 250 mm semi-prep RP-HPLC column equilibrated with 10 % MeCN + 0.1% TFA and peptides were eluted with a 20 column volume gradient to 90 % MeCN + 0.1% TFA. BRC repeat peptides typically elute at 20-40% of the gradient. Peak fractions corresponding to the cleaved peptide were pooled and diluted 5x in PBS + 10 mM EDTA in a 50 ml centrifuge tube. A small stirrer bar was added to the tube, which was then placed on a magnetic stirrer. A syringe was filled with 20 mM linker in DMSO which was then gradually added to the mixture by piercing the centrifuge tube lid. To maintain pseudo-dilution conditions, linker was injected in 50 µl increments every two minutes, to a final concentration of 2 mM, ensuring at least 2x stoichiometric excess of linker over peptide. The reaction mixture was then quenched with 5 mM TCEP, filtered through a 0.45 µm filter and acidified with HCl to pH ~3. Stapled peptide was then purified by a on an ACE C18 300 4.6 x 250 mm semi-prep RP-HPLC column with a 0-100% gradient of A: 10 % MeCN + 0.1% TFA, B: 90 % MeCN + 0.1% TFA. Peak fractions containing the desired product were pooled and dried under vacuum.

Purification of HumRadA22

HumRadA22 is an archaeal RadA mutant with surface residues exchanged for the human Rad51 sequence and can be used as a bona fide mimic of monomeric human Rad51. The protein was prepared as described previously.[2]

Purification of full-length human RAD51

Full-length HsRAD51 was prepared based on a protocol developed at the lab of Prof Luca Pellegrini (Department of Biochemistry, University of Cambridge). *E. coli* BL21(DE3) Rosetta2 cells (Novagen) carrying a pRSF-Duet plasmid co-expressing wild-type *Hs*RAD51 and a BRC4 sequence fused to an N-terminal His-MBP tag were kindly provided by Dr Joseph

Maman. Cells were plated from a glycerol stock on LB agar supplemented with kanamycin (25 μg/mL) and chloramphenicol (34 μg/mL), and grown overnight at 37°C. Next day, cells were scraped and used to inoculate 1 L of 2xYT medium supplemented with same antibiotics. Cells were grown at 37°C with shaking at 200 RPM until an OD600 = 0.6, afer which they were cooled down to 18°C and expression induced with IPTG (400 μM) overnight. Cells were resuspended in 25 mL of buffer Ni-A-300 (50 mM Tris-HCl, pH 8.0, 300 mM NaCl, 20 mM imidazole, 1 mM TCEP) and frozen. Later, cells were thawed and supplemented with DNase I (100 μL, 2 mg/mL) and AEBSF (1 mM), and lysed on an Emulsiflex C5 homogenizer (Avestin) or by sonication. Cell lysate was spun down at 40 000 g for 30 min, after which the soluble fraction was loaded on a HisTrap HP 5 ml column (Cytiva). The column was washed with 8 CV Ni-A300 buffer, after which MBP-BRC4:RAD51 complex was co-eluted with buffer Ni-B-300 (50 mM Tris-HCl, pH 8.0, 300 mM NaCl, 200 mM imidazole, 1 mM TCEP). The sample was then diluted with Heparin-A buffer (20 mM HEPES pH 7.4, 50 mM NaCl, 1 mM EDTA, 1 mM TCEP) and loaded on a HiTrap Heparin HP 5 ml column. During this step, RAD51 oligomerises on the heparin matrix, which acts as a DNA mimic, and dissociates from the MBP-BRC4 fusion, which is removed in flow-through and wash steps. Column was washed with 8 CV Heparin-A buffer, after which the protein was eluted with a 20 CV, 0-100% linear gradient of Heparin-B buffer (20 mM HEPES pH 7.4, 1 M NaCl, 1 mM EDTA, 1 mM TCEP). RAD51 was concentrated, flash-frozen with liquid nitrogen and stored for future use.

Fluorescence polarisation competition assay

FP competition assay was a modified version of a protocol described previously.[2] Fluorescein-labelled *Hs*BRC4 probe for this assay was kindly provided by Dr Laurens Lindenburg (Hollfelder group, Department of Biochemistry, University of Cambridge). Black 384-well flat-bottom microplates (Corning, 3821) were used with a 40 μl final reaction volume in all measurements. Following buffer conditions were used: 20 mM CHES pH 9.5, 150 mM NaCl, 0.1% BSA, 0.1% Tween-20. Each reaction contained 100 nM HumRadA22 and 10 nM BRC4-fluorescein. Two-fold serial dilutions of stapled peptides were added to the reactions. A free probe control reaction containing only 10 nM BRC4-fluorescein was used to calibrate gain and focal height. FP measurements were performed on Pherastar FX or Clariostar Plus (BMG Labtech) plate readers equipped with an FP 485-520-520 optic modules. Binding curves were fitted using the four-parameter logistic model with a variable Hill slope using Prism software (Graphpad). Regression fitting was performed using the least squares optimisation algorithm. K_D values were estimated from the fitted IC_{50} parameters using a previously reported equation.[6]

Isothermal titration calorimetry

Peptides were resuspended in MilliQ water to 10 times the desired final concentration. This was then diluted 10x with the ITC buffer to obtain the final titrant solution (20 mM CHES pH 9.5, 150 mM NaCl, 0.1% Tween-20). HumRadA22 was buffer-exchanged on a NAP-5 desalting column (Cytiva) into ITC buffer and protein concentration was adjusted to 10:9 of the desired final value. One ninth volume of MilliQ water was added to the solution to bring the protein concentration to the desired final value, while maintaining identical buffer:MilliQ volume proportions in both the syringe and the cell. ITC was carried out using a Microcal ITC200 or Malvern PEAQ ITC instruments at 25°C with a 5.00 µCal reference power DP value, stirring speed of 500-750 rpm, 2 sec filter period. Injection spacing, speed and volume, cell/syringe concentrations as well as the number of injections were adjusted for each peptide and its binding properties. ITC data were fitted using a single-site binding model using the Microcal ITC data analysis program in the Origin 7.0 package. Data points affected by baseline spikes were omitted from the analysis.

Circular dichroism spectroscopy

Dried peptides were dissolved in MilliQ water to 0.3 mg/ml, and then two-fold diluted in 20 mM sodium phosphate, pH 7.4, giving a final solution of 0.15 mg/ml peptide in 10 mM sodium phosphate. CD spectra of selected peptides were recorded on an AVIV 410 circular dichroism spectropolarimeter using a 1 mm path length quartz cuvette. Measurements were done at 25 °C, with a 185-260 nm range, 1 nm bandwidth, 5 s averaging time and 0.3 s settling time. Spectra were prepared as smoothed average of three scans and normalised against blank solvent.

Electrophoretic mobility shift assay (EMSA)

The ability of linear and stapled BRC repeat peptides to dissociate RAD51-ssDNA nucleofilament was evaluated using an electrophoretic mobility shift assay (EMSA). RAD51 DNA-binding reactions (40 µl) were set up in 50 mM HEPES pH 7.4, 150 mM NaCl, 10 mM MgAc2, 2 mM CaCl2, 1 mM TCEP, 1 mM ATP. 5 µM full-length human RAD51 was incubated with varying concentrations of BRC repeats for 10 min at room temperature, followed by the addition of 100 nM fluorescently labelled FAM-dT60 oligonucleotide, and further incubation at 37 °C for 10 min. Control reactions were set up with free FAM-dT60 probe and FAM-dT60 Pantelejevs, T. Materials and methods 120 + 5 µM RAD51. 10 µl of reactions were then loaded on a 1xTBE non-denaturing acrylamide gel (5%) and run at 100 V for 1:30 h at 4 °C. The gel was directly visualized on a Typhoon FLA 9000 imager (GE Healthcare) using FAM channels.

X-ray crystallography

Stapled peptide complexes were re-constituted from purified peptides and HumRadA22. Peptides were added at a 1.5 stoichiometric excess to HumRadA22 in its size-exclusion buffer, to a final concentration of 0.75 and 0.5 mM for the peptide and protein, respectively. ADP and $MgCl_2$ were added to the protein solutions in for some of the complexes (see **Table S1**). Crystallisation screening was done in 96-well MRC plates using the sitting-drop vapor diffusion technique and a variety of commercial crystallisation screens. A Mosquito liquid handling robot (TTP Labtech) was used to dispense protein and reservoir solutions in sub-microlitre volumes. Typically the two sitting drops contained 200 or 400 nl of protein solution and 200 nl of crystallisation solution, while the reservoir contained 80 µl of crystallisation solution. Plates were stored at 17 °C in a RockImager crystallisation hotel (Formulatrix) and imaged regularly. Crystal hits were flash-frozen in liquid nitrogen using cryoloops. Additional cryoprotectant was not added before freezing of crystal hits. Diffraction data were collected on Diamond Light Source (Harwell, UK) MX beamlines. Full native datasets with goniometer sweeps of at least 180° were collected to ensure completeness of diffraction data. Molecular replacement phasing method was used with the apo HumRadA22 structure (PDB: 5KDD) as a search model. Molecular replacement was done with Phaser.[7] The structures were refined without BRC repeats first and the peptides were built into the clearly visible electron density manually. Manual refinement was done in Coot[8] and automated refinement with phenix.refine[7] and autoBUSTER.[9] Crystallisation conditions, as well as data collection and refinement statistics, are provided in **Table S1**. The coordinates have been deposited in the Protein Data Bank under accession codes 8C3J (**SP2**), 8BR9 (**SP24**) and 8C3N (**SP30**).

Cell line

U2OS cell line (ATCC, HTB-96) was used in this study. They were cultured in DMEM medium (Sigma-Aldrich, D6429) containing 10% fetal bovine serum (Sigma-Aldrich, F7524) and 100 U/mL penicillin/streptomycin (Sigma-Aldrich, P4333) at 37 °C and 5% CO2

Immunofluorescence

Between 12000-15000 cells per well were seeded in 96-well plates (PerkinElmer, 6055302). After 24 hours, medium was replaced by fresh medium containing or not **SP30** or **SP31** or **L31** (40 µM). Cells were incubated for an hour before they were irradiated (3 Gy) and allowed to recover for 3 hours before being fixed. Non-irradiated cells treated in a similar way were used as a control. RAD51 foci were detected following a protocol previously described.[1] In summary, after being washed with PBS, cells were fixed with paraformaldehyde, 4% in PBS

(freshly made from paraformaldehyde 32% Aqueous sol. EM GRADE, Electron Microscopy Science, 15714-S) for 15 minutes at room temperature. After washing with PBS, cells were permeabilised with Triton X-100 (Sigma-Aldrich, T8787), 0.5% in PBS for 7 min at room temperature. Cells were washed again and blocked with 1% BSA (Sigma-Aldrich, A9647) in PBS for at least an hour at room temperature. RAD51 antibody (Abcam, ab63801) was diluted 1:10000 in blocking solution and added to the fixed cells at 4 °C overnight. After washing with PBS, cells were incubated with anti-rabbit IgG Alexa Fluor 488 (Life Technologies, A21206) diluted 1:500 in the blocking solution for around an hour at room temperature. Finally, cells were washed with PBS, incubated with Hoechst 33342 (Invitrogen, H1399) 10 µg/mL in blocking solution for 2 minutes at room temperature and washed again with PBS. Cells were kept in PBS at 4 °C until images were taken using an Opera Phenix Plus High-Content Screening System (Perkin Elmer) with a 40x objective and Harmony High-Content Imaging and Analysis Software (version 5.1). Between 3500 and 5500 cells were analysed by condition and experiment. RAD51 foci were counted using SImA (version 1.2.29, Signals Image Artist, Perkin Elmer), ImageJ (version 1.54f) and GraphPad Prism (version 9.4.1).

X-ray crystallography statistics

Complex	SP2:HumRadA22	SP24:HumRadA22	SP30:HumRadA22
**Protein**	0.5 mM SP2:HumRadA22 in 20 mM CHES pH 9.5, 100 mM NaCl	0.5 mM SP24:HumRadA22 in 20 mM CHES pH 9.5, 100 mM NaCl, 20 mM ADP/$MgCl_2$	0.5 mM SP30:HumRadA22 in 20 mM CHES pH 9.5, 100 mM NaCl, 20 mM ADP/$MgCl_2$
**Condition**	0.1 M Na_3Citrate pH 4.2, 20% w/v PEG 1K, 0.2 M Li_2SO_4	14% w/v PEG 4000, 6% v/v MPD, 0.1M Na K Phos pH 6.2	14% w/v PEG 4000, 6% v/v MPD, 0.1M Na K Phos pH 6.2
**Protein:well solution (nl:nl)**	200:200	400:200	200:200
**PDB code**	8C3J	8BR9	8C3N
**Data collection and processing**			
Beamline	DLS i03	DLS i04	DLS i04
Wavelength (Å)	0.9762	0.9795	0.9795
Space group	P 41 21 2	P 21 21 2	P 21 21 2
a, b, c (Å)	112.56 112.56 140.79	140.46 38.64 43.53	143.13 38.01 43.92
α, β, γ (°)	90.00 90.00 90.00	90.00 90.00 90.00	90.00 90.00 90.00
Resolution range (Å)	87.92 - 3.02 (3.02 - 3.07)	70.23 - 1.63 (1.66 - 1.63)	1.25 - 41.99 (1.25 - 1.27)
R_{meas}	0.242 (3.751)	0.059 (4.504)	0.046 (1.597)
Completeness (%)	100.0 (100.0)	98.7 (99.2)	99.6 (95.3)
Reflections	170095 / 13512	239726 / 30303	513195 / 67481
Redundancy	12.8 (13.8)	7.9 (7.8)	7.6 (4.6)
$<I/\sigma(I)>$	10.0 (0.9)	12.0 (0.3)	15.8 (0.8)
CC½	1.0 (0.3)	1.0 (0.3)	1.0 (0.4)
**Refinemnt**			
R_{cryst}/R_{free}	0.228 / 0.254	0.260 / 0.270	0.196 / 0.210
Resolution range (Å)	69.29 - 3.02	70.23 - 1.62	36.74 - 1.24
Reflections in work / test set	17515 / 897	27946 / 1450	64107 / 3361
Number of atoms	3834	2041	2373
Mean / Wilson B-factor ($Å^2$)	95.6 / 79.5	63.3 / 34.4	27.9 / 17.8
Ramachandran favoured/allowed/outliers (%)	97.22 / 2.57 / 0.21	99.17 / 0.83 / 0.00	99.15 / 0.85 / 0.00
RMSD bonds (Å)	0.017	0.013	0.014
RMSD angles (°)	1.66	1.69	1.61

Table S1. Crystallographic data collection and refinement. Values in parentheses are for the high-resolution cell.

Oligonucleotides used for cloning of peptides

Insert	Vector	Res. enzyme(s)	No	Sequence
SP1	pPEPT1	*BsaI*	1	TTCCAGGGATCCGACCCTTCTTGTGAAGCGCTGCAGAAGGCGTGTAAACTGTTCTC
			2	ATGGCTCGAGCCAGGGTCAGAGATGTTTTCGATGTCGGAGAACAGTTTACACGCCTT
SP2	pOP3BT	*BamHI/XhoI*	1	CCTGTACTTCCAGGGATCCGTTAACTCTTCTGCGTTCTCCG
			2	CAGTTTTTTACCAGACGCGGTAGAGAAACCGGAGAACGCAGAAGAGTTAA
			3	CCGCGTCTGGTAAAAAACTGAACGTTTCTTGCGAAGCGCTGCAGAAAGCG
			4	CAAGCTTAGCTCGAGCCAGAGATGTTTTCGATGTCAGAGAACAGTTTGCACGCTTTCTGCAGCGCTT
	pPEPT1	*BsaI*	1	TTCCAGGGATCCGACCCTGTTAACTCTTCTGCGTTCTCCGGTTTCTCTACCGCGTCTGGTAAAAAACTGAACGTTTCTTGCGA
			2	ATGGCTCGAGCCAGGGTCAGAGATGTTTTCGATGTCAGAGAACAGTTTGCACGCTTTCTGCAGCGCTTCGCAAGAAACGTTCAGTTTT
SP7	pPEPT1	*BsaI*	1	TTCCAGGGATCCGACCCTGTTAACTCTTCTGCGTTCTCTGGTTTCTGCACCGCGTCTGGTAAAAAACTGAACGTTTCTTGCGA
			2	ATGGCTCGAGCCAGGGTCAGAGATGTTTTCGATGTCGGAGAACAGTTTAACCGCCTTCTGCAGCGCTTCGCAAGAAACGTTCAGTTTT
SP8	pPEPT1	*BsaI*	1	TTCCAGGGATCCGACCCTGTTAACTCTTCTGCGTTCTCTGGTTTCTCTACCGCGTCTGGTAAAAAACTGAACGTTTCTACCTGTG
			2	ATGGCTCGAGCCAGGGTCAGAGATGTTTTCGATGTCGGAGAACAGACAAACCGCCTTCTGCAGCGCACAGGTAGAAACGTTCAGT
SP9	pPEPT1	*BsaI*	1	TTCCAGGGATCCGACCCTGTTAACTCTTCTGCGTTCTCTGGTTTCTCTACCGCGTCTGGTAAAAAACTGAACGTTTCTACCGAAG
			2	ATGGCTCGAGCCAGGGTCAGAGATGTTTTCGATGTCACAGAACAGTTTAACCGCCTTACACAGCGCTTCGGTAGAAACGTTCAGT
SP10	pPEPT1	*BsaI*	1	TTCCAGGGATCCGACCCTGCGTGTGGTTTCTCTACCGCGTCTGGTAAAAAACTGAACGTTTCTTGTGAAGCGCTGCAG
			2	ATGGCTCGAGCCAGGGTCAGAGATGTTTTCGATGTCGGAGAACAGTTTAACCGCCTTCTGCAGCGCTTCACAAGAAA
SP11	pPEPT1	*BsaI*	1	TTCCAGGGATCCGACCCTTCTTGTTTCTCTACCGCGTCTGGTAAAAAACTGAACGTTTCTTGTGAAGCGCTGCAGA
			2	ATGGCTCGAGCCAGGGTCAGAGATGTTTTCGATGTCGGAGAACAGTTTAACCGCCTTCTGCAGCGCTTCACAAGAA
SP12	pPEPT1	*BsaI*	1	TTCCAGGGATCCGACCCTTCTTCTGCGTGTTCTGGTTTCTCTACCGCGTCTGGTAAAAAACTGAACGTTTCTACCGAAGCGT
			2	ATGGCTCGAGCCAGGGTCAGAGATGTTTTCGATGTCGGAGAACAGTTTAACCGCCTTCTGACACGCTTCGGTAGAAACGTTC
SP13	pPEPT1	*BsaI*	1	TTCCAGGGATCCGACCCTGTTAACTGTTCTGCGTTCTCTGGTTTCTCTACCGCGTCTGGTAAAAAACTGAACGTTTCTACCGAAG
			2	ATGGCTCGAGCCAGGGTCAGAGATGTTTTCGATGTCGGAGAACAGTTTAACCGCCTTACACAGCGCTTCGGTAGAAACGTTCAGT
SP14	pPEPT1	*BsaI*	1	TTCCAGGGATCCGACCCTGTTAACTGTTCTGCGTTCTCTGGTTTCTCTACCGCGTCTG
			2	ATGGCTCGAGCCAGGGTCAGAAACACACAGTTTTTTACCAGACGCGGTAGAGAAACCA
SP16	pPEPT1	*BsaI*	1	TTCCAGGGATCCGACCCTGCGTGTTCTGGTTTCTCTACCGCGTCTGGTAAAAA
			2	ATGGCTCGAGCCAGGGTCAGAACAAACGTTCAGTTTTTTACCAGACGCGGTAGA
SP24	pEXP-Nhis-GB1	*BsaI/HindIII*	1	ACCTGTACTTCCAGGGATCCGGTTTCTCTACCGCGTCTGGT
			2	GCAGCGCCTGGCAAGAAACGTTCAGTTTTTTACCAGACGCGGTAGAGAAA
			3	CTTGCCAGGCGCTGCAGAAAGCGTGCAAACTGTTCTCTGGTAGCCATCAT
			4	TGGTGATGGTGATGGCTCGAGCCCCAATGATGGCTACCAGAGAACAG
SP30	pEXP-Nhis-GB1	*BsaI/HindIII*	1	TTCTAATACGACTCACTATAGGTACCGAAAACCTGTACTTCC
			2	GCCGCTCGCGGTGCTAAAGCCGCTGCAGCCCTGGAAGTACAGGTTTTCGGTACCTATAGTGAGTC
			3	CCGCGAGCGGCAAAAAACTGAACGTGAGCACCCAGGCGTGCCAGA
			4	CTATAGAATACTCAAGCTTAGCCGCTAAACAGTTTCACCGCTTTCTGGCACGCCTGGGT

Table S2. Oligonucleotides used for the cloning of peptide constructs.

pPEPT1 vector

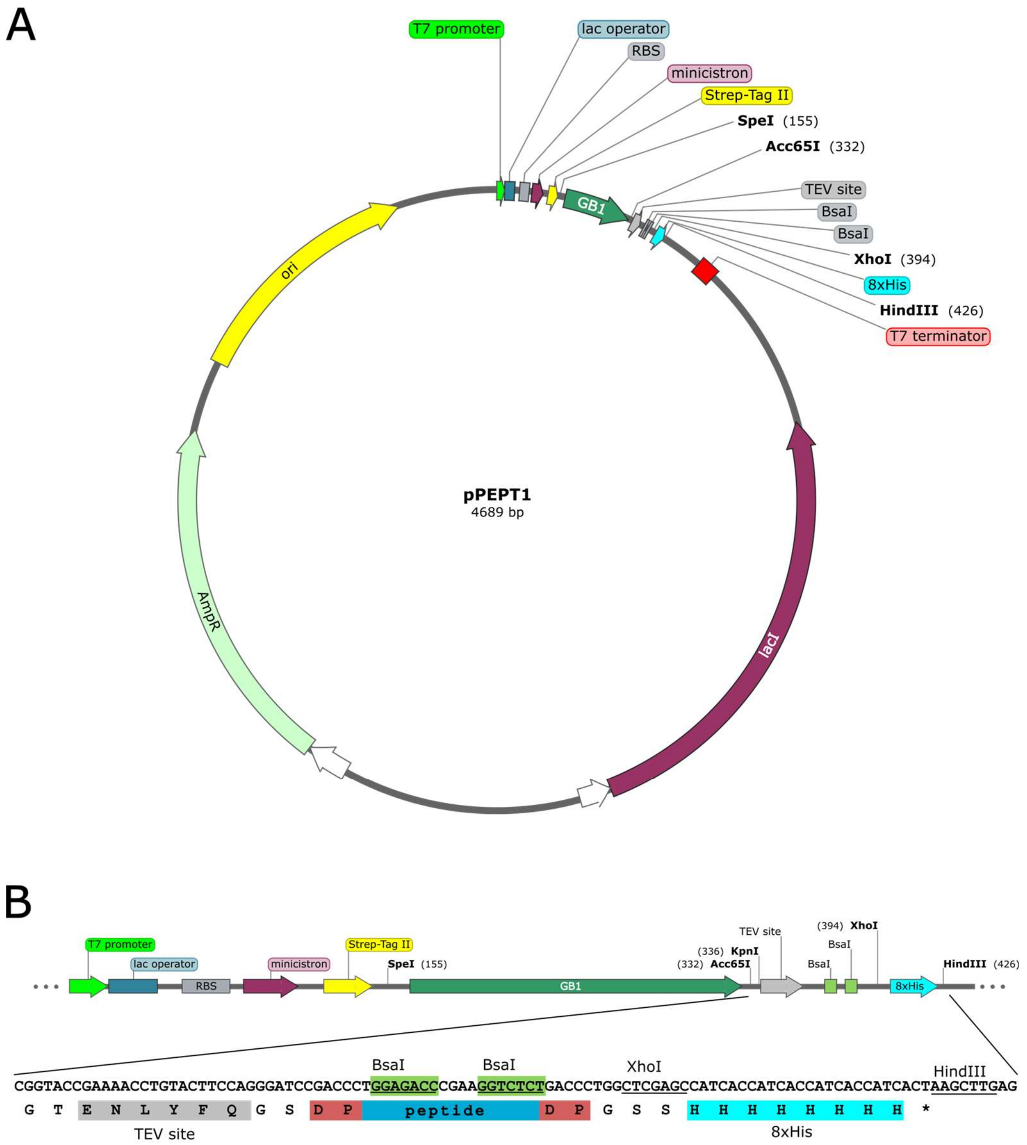

Figure S1. Map of the pPEPT1 plasmid. (A) circular plasmid map of the pPEPT1 vector with key features and unique restrictions sites in the fusion part and in the multiple cloning site. (B) Focused view of the DNA and protein features of the part where the peptide-encoding sequences are inserted.

Optimisation of the small-scale stapling reaction

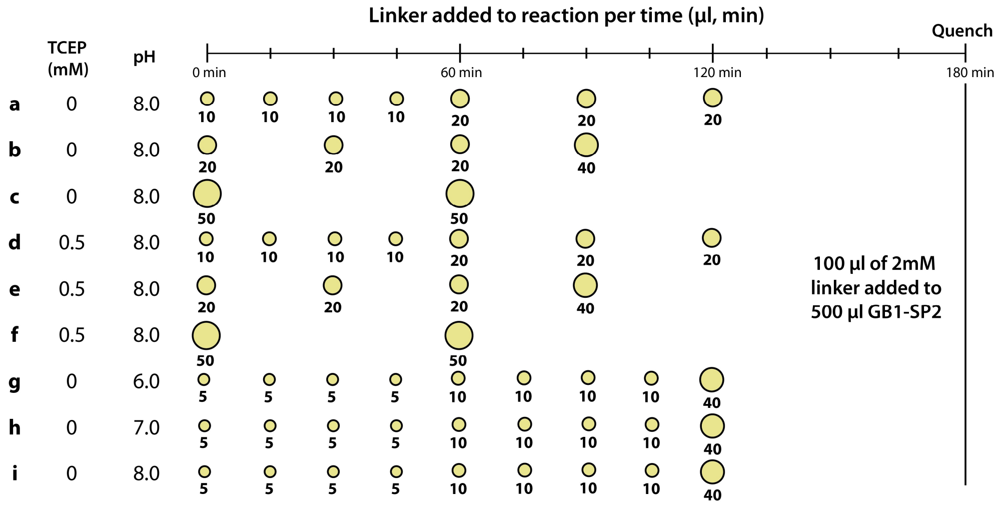

Figure S2. Optimisation of small-scale stapling reaction. Lower-case letters a-i represent different conditions. For each condition, the volumes (µl) of linker addition are depicted at appropriate time-points under the yellow circles, are which is relative to the volume for more visual interpretation of the experiments. pH and presence of TCEP are also indicated. ESI-MS mass spectra for each condition are shown below.

SP31 *in vitro* binding data to HumRadA22

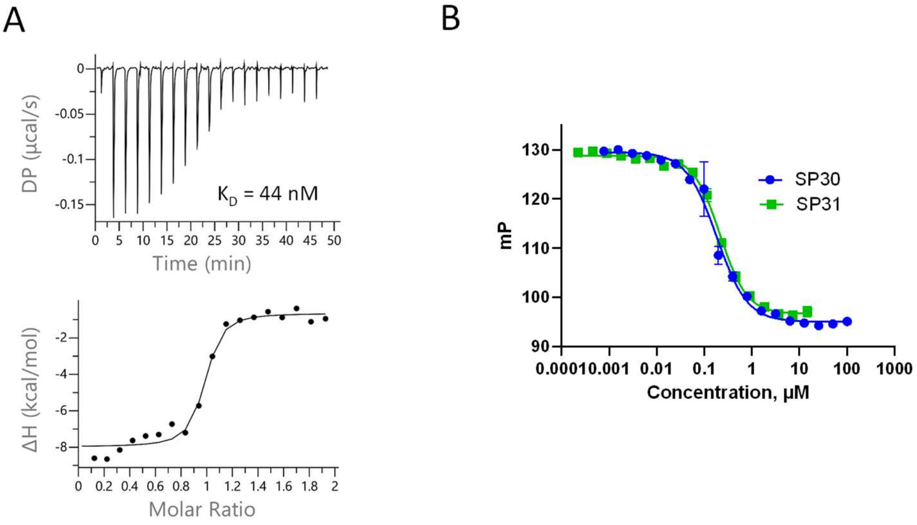

Figure S3. (**A**) ITC and (**B**) fluorescence polarisation competition measurements demonstrating that poly-arginine containing **SP31** similarly to **SP30** has high *in vitro* binding affinity for HumRadA22.

Rad51 foci inhibition data for stapled peptides SP30 and SP31

A

Experiment 1

Control noIR	No cells	%
Foci = 0	1160	32.7
Foci = 1	507	14.3
Foci = 2	304	8.6
Foci = 3	246	6.9
Foci = 4	178	5.0
Foci 5-9	745	21.0
Foci > 10	409	11.5
	3549	100

Stpl30 noIR	No cells	%
Foci = 0	1299	30.9
Foci = 1	590	14.0
Foci = 2	333	7.9
Foci = 3	260	6.2
Foci = 4	235	5.6
Foci 5-9	887	21.1
Foci > 10	606	14.4
	4210	100

Stpl31 noIR	No cells	%
Foci = 0	2001	38.2
Foci = 1	867	16.5
Foci = 2	496	9.5
Foci = 3	333	6.4
Foci = 4	280	5.3
Foci 5-9	855	16.3
Foci > 10	409	7.8
	5241	100

Control 3Gy 3h	No cells	%
Foci = 0	529	13.1
Foci = 1	370	9.2
Foci = 2	241	6.0
Foci = 3	215	5.3
Foci = 4	159	3.9
Foci 5-9	599	14.9
Foci > 10	1914	47.5
	4027	100

Stpl30 3Gy 3h	No cells	%
Foci = 0	539	12.3
Foci = 1	356	8.1
Foci = 2	287	6.5
Foci = 3	235	5.4
Foci = 4	165	3.8
Foci 5-9	621	14.2
Foci > 10	2180	49.7
	4383	100

Stpl31 3Gy 3h	No cells	%
Foci = 0	1334	24.6
Foci = 1	715	13.2
Foci = 2	422	7.8
Foci = 3	333	6.1
Foci = 4	238	4.4
Foci 5-9	877	16.2
Foci > 10	1498	27.7
	5417	100

Experiment 2

Control noIR	No cells	%
Foci = 0	1630	34.5
Foci = 1	748	15.8
Foci = 2	392	8.3
Foci = 3	297	6.3
Foci = 4	271	5.7
Foci 5-9	915	19.3
Foci > 10	478	10.1
	4731	100

Stpl30 noIR	No cells	%
Foci = 0	1623	33.5
Foci = 1	769	15.9
Foci = 2	414	8.5
Foci = 3	304	6.3
Foci = 4	227	4.7
Foci 5-9	891	18.4
Foci > 10	623	12.8
	4851	100

Stpl31 noIR	No cells	%
Foci = 0	1799	37.7
Foci = 1	792	16.6
Foci = 2	442	9.3
Foci = 3	306	6.4
Foci = 4	258	5.4
Foci 5-9	786	16.5
Foci > 10	384	8.1
	4767	100

Control 3Gy 3h	No cells	%
Foci = 0	805	17.6
Foci = 1	534	11.7
Foci = 2	314	6.9
Foci = 3	197	4.3
Foci = 4	183	4.0
Foci 5-9	598	13.1
Foci > 10	1943	42.5
	4574	100

Stpl30 3Gy 3h	No cells	%
Foci = 0	846	17.2
Foci = 1	545	11.1
Foci = 2	315	6.4
Foci = 3	218	4.4
Foci = 4	157	3.2
Foci 5-9	617	12.6
Foci > 10	2209	45.0
	4907	100

Stpl31 3Gy 3h	No cells	%
Foci = 0	943	20.3
Foci = 1	532	11.5
Foci = 2	361	7.8
Foci = 3	235	5.1
Foci = 4	174	3.7
Foci 5-9	702	15.1
Foci > 10	1695	36.5
	4642	100

Experiment 3

Control noIR	No cells	%
Foci = 0	1313	33.6
Foci = 1	463	11.8
Foci = 2	295	7.5
Foci = 3	230	5.9
Foci = 4	202	5.2
Foci 5-9	819	21.0
Foci > 10	586	15.0
	3908	100

Stpl30 noIR	No cells	%
Foci = 0	1537	38.4
Foci = 1	406	10.2
Foci = 2	263	6.6
Foci = 3	253	6.3
Foci = 4	227	5.7
Foci 5-9	869	21.7
Foci > 10	443	11.1
	3998	100

Stpl31 noIR	No cells	%
Foci = 0	1869	44.1
Foci = 1	593	14.0
Foci = 2	348	8.2
Foci = 3	262	6.2
Foci = 4	232	5.5
Foci 5-9	665	15.7
Foci > 10	273	6.4
	4242	100

Control 3Gy 3h	No cells	%
Foci = 0	876	23.8
Foci = 1	344	9.3
Foci = 2	200	5.4
Foci = 3	149	4.0
Foci = 4	124	3.4
Foci 5-9	612	16.6
Foci > 10	1381	37.5
	3686	100

Stpl30 3Gy 3h	No cells	%
Foci = 0	913	20.9
Foci = 1	390	8.9
Foci = 2	217	5.0
Foci = 3	159	3.6
Foci = 4	138	3.2
Foci 5-9	691	15.8
Foci > 10	1852	42.5
	4360	100

Stpl31 3Gy 3h	No cells	%
Foci = 0	1343	29.9
Foci = 1	518	11.5
Foci = 2	328	7.3
Foci = 3	225	5.0
Foci = 4	195	4.3
Foci 5-9	729	16.2
Foci > 10	1155	25.7
	4493	100

Experiment 4

Control noIR	No cells	%
Foci = 0	1331	28.5
Foci = 1	540	11.6
Foci = 2	324	6.9
Foci – 3	258	5.5
Foci = 4	252	5.4
Foci 5-9	1123	24.1
Foci > 10	836	17.9
	4664	100

Stpl30 noIR	No cells	%
Foci = 0	1442	31.3
Foci = 1	510	11.1
Foci = 2	296	6.4
Foci = 3	279	6.1
Foci = 4	253	5.5
Foci 5-9	1036	22.5
Foci > 10	789	17.1
	4605	100

Stpl31 noIR	No cells	%
Foci = 0	2012	41.5
Foci = 1	964	19.9
Foci = 2	536	11.1
Foci = 3	359	7.4
Foci = 4	284	5.9
Foci 5-9	574	11.8
Foci > 10	117	2.4
	4846	100

Control 3Gy 3h	No cells	%
Foci = 0	783	17.4
Foci = 1	382	8.5
Foci = 2	253	5.6
Foci = 3	207	4.6
Foci = 4	186	4.1
Foci 5-9	863	19.2
Foci > 10	1832	40.7
	4506	100

Stpl30 3Gy 3h	No cells	%
Foci = 0	704	14.4
Foci = 1	467	9.6
Foci = 2	253	5.2
Foci = 3	252	5.2
Foci = 4	178	3.6
Foci 5-9	835	17.1
Foci > 10	2188	44.9
	4877	100

Stpl31 3Gy 3h	No cells	%
Foci = 0	1590	31.9
Foci = 1	953	19.1
Foci = 2	632	12.7
Foci – 3	350	7.0
Foci = 4	258	5.2
Foci 5-9	667	13.4
Foci > 10	541	10.8
	4991	100

Experiment 5

Control noIR	No cells	%
Foci = 0	1358	26.7
Foci = 1	586	11.5
Foci = 2	337	6.6
Foci = 3	274	5.4
Foci = 4	204	4.0
Foci 5-9	1250	24.6
Foci > 10	1081	21.2
	5090	100

Stpl30 noIR	No cells	%
Foci = 0	1421	27.2
Foci = 1	664	12.7
Foci = 2	329	6.3
Foci = 3	270	5.2
Foci = 4	238	4.5
Foci 5-9	1210	23.1
Foci > 10	1101	21.0
	5233	100

Stpl31 noIR	No cells	%
Foci = 0	1566	32.1
Foci = 1	778	15.9
Foci = 2	473	9.7
Foci = 3	343	7.0
Foci = 4	336	6.9
Foci 5-9	957	19.6
Foci > 10	431	8.8
	4884	100

Control 3Gy 3h	No cells	%
Foci = 0	741	14.2
Foci = 1	445	8.5
Foci = 2	318	6.1
Foci = 3	215	4.1
Foci = 4	164	3.1
Foci 5-9	743	14.2
Foci > 10	2596	49.7
	5222	100

Stpl30 3Gy 3h	No cells	%
Foci = 0	672	13.1
Foci = 1	382	7.4
Foci = 2	305	5.9
Foci = 3	217	4.2
Foci = 4	169	3.3
Foci 5-9	730	14.2
Foci > 10	2664	51.8
	5139	100

Stpl31 3Gy 3h	No cells	%
Foci = 0	1106	23.0
Foci = 1	685	14.2
Foci = 2	437	9.1
Foci = 3	309	6.4
Foci = 4	252	5.2
Foci 5-9	781	16.2
Foci > 10	1241	25.8
	4811	100

B

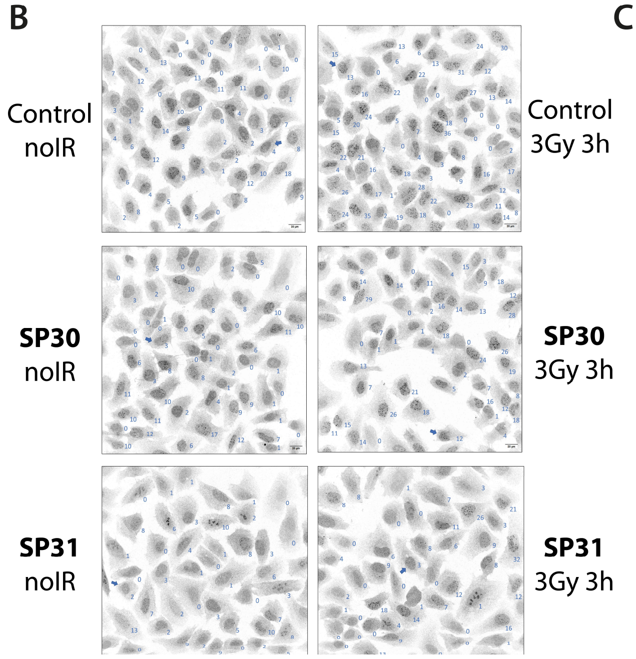

C

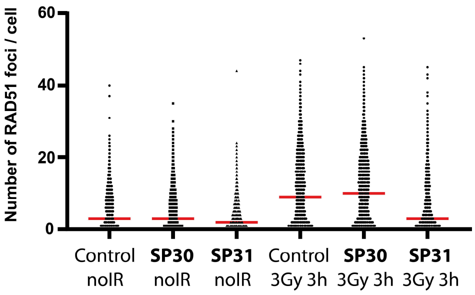

Figure S4. (**A**) Quantification of RAD51 foci in U2OS cells from five independent experiments in the presence of 40 μM **SP30**, **SP31** or vehicle (control). (**B**) Representative IF images depicting RAD51 signal. Representative cells shown in **Figure 5A** are indicated with an arrow and they were chosen as the median RAD51 foci value for each of the images. Foci counts determined automatically using SImA software are provided next to each cell. Scale bar 20 μm. (**C**) Dot plot depicting the number of RAD51 foci per cell for one of the independent biological experiments. Red bars represent the mean values.

Rad51 foci inhibition data for linear peptide L31

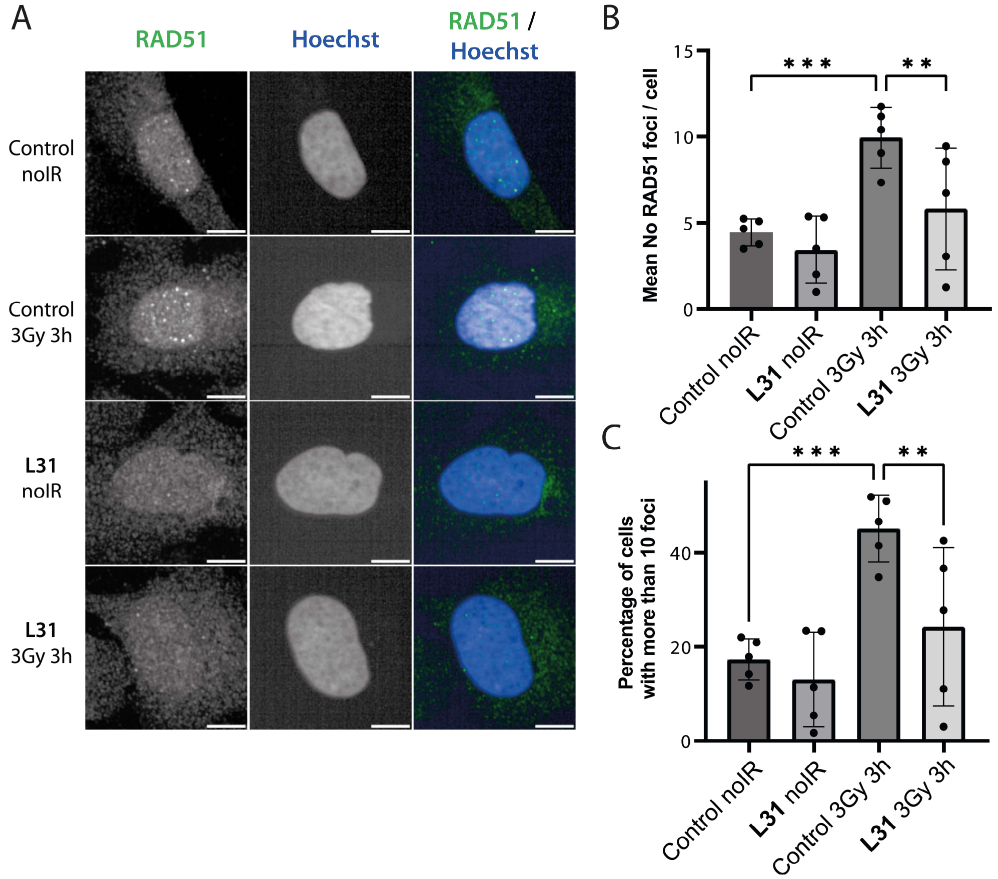

Figure S5. (**A**) Representative immunofluorescence images of U2OS cells incubated with linear peptide **L31** (40 µM, L31) or vehicle alone (Control) for 1 hour, after which they were treated with 3Gy ionising radiation (3Gy 3h) or no radiation (noIR) and allowed to recover for 3 hours. Cells were stained with α-RAD51 and Hoechst 33342 as indicated. Scale bar is 10 µm. (**B**) Bar graph showing the average of the mean counts of RAD51 foci per cell from five independent biological experiments. Data are presented as mean ± SD, ** - $P < 0.01$, ***- $P < 0.001$, using ANOVA test followed by Tukey's method. (**C**) Bar graph showing the average percentage of U2OS cells with more than 10 RAD51 foci per cell. Data are presented as mean ± SD, ** - $P < 0.01$, ***- $P < 0.001$, using ANOVA test followed by Tukey's method.

A

Experiment 1

Control no IR	No cells	%	L31 noIR	No cells	%	Control 3Gy 3h	No cells	%	L31 3Gy 3h	No cells	%
Foci = 0	1672	40.3	Foci = 0	3076	62.5	Foci = 0	1006	22.9	Foci = 0	2853	59.4
Foci = 1	407	9.8	Foci = 1	980	19.9	Foci = 1	378	8.6	Foci = 1	1005	20.9
Foci = 2	287	6.9	Foci = 2	338	6.9	Foci = 2	216	4.9	Foci = 2	344	7.2
Foci = 3	226	5.4	Foci = 3	149	3.0	Foci = 3	172	3.9	Foci = 3	184	3.8
Foci = 4	196	4.7	Foci = 4	85	1.7	Foci = 4	138	3.1	Foci = 4	91	1.9
Foci 5-9	881	21.2	Foci 5-9	209	4.2	Foci 5-9	665	15.1	Foci 5-9	187	3.9
Foci > 10	485	11.7	Foci > 10	82	1.7	Foci > 10	1824	41.5	Foci > 10	143	3.0
	4154	100		4919	100		4399	100		4807	100

Experiment 2

Control no IR	No cells	%	L31 noIR	No cells	%	Control 3Gy 3h	No cells	%	L31 3Gy 3h	No cells	%
Foci = 0	1397	27.6	Foci = 0	1374	27.3	Foci = 0	665	13.5	Foci = 0	654	13.9
Foci = 1	516	10.2	Foci = 1	611	12.1	Foci = 1	405	8.2	Foci = 1	502	10.7
Foci = 2	307	6.1	Foci = 2	359	7.1	Foci = 2	288	5.8	Foci = 2	324	6.9
Foci = 3	286	5.7	Foci = 3	266	5.3	Foci = 3	229	4.6	Foci = 3	254	5.4
Foci = 4	255	5.0	Foci = 4	242	4.8	Foci = 4	197	4.0	Foci = 4	190	4.0
Foci 5-9	1182	23.4	Foci 5-9	1002	19.9	Foci 5-9	851	17.2	Foci 5-9	779	16.5
Foci > 10	1110	22.0	Foci > 10	1175	23.4	Foci > 10	2308	46.7	Foci > 10	2007	42.6
	5053	100		5029	100		4943	100		4710	100

Experiment 3

Control no IR	No cells	%	L31 noIR	No cells	%	Control 3Gy 3h	No cells	%	L31 3Gy 3h	No cells	%
Foci = 0	1447	27.3	Foci = 0	1196	24.7	Foci = 0	695	13.1	Foci = 0	800	17.1
Foci = 1	641	12.1	Foci = 1	727	15.0	Foci = 1	442	8.4	Foci = 1	592	12.7
Foci = 2	356	6.7	Foci = 2	427	8.8	Foci = 2	314	5.9	Foci = 2	425	9.1
Foci = 3	250	4.7	Foci = 3	284	5.9	Foci = 3	232	4.4	Foci = 3	257	5.5
Foci = 4	232	4.4	Foci = 4	232	4.8	Foci = 4	179	3.4	Foci = 4	201	4.3
Foci 5-9	1260	23.8	Foci 5-9	849	17.5	Foci 5-9	728	13.8	Foci 5-9	680	14.6
Foci > 10	1108	20.9	Foci > 10	1129	23.3	Foci > 10	2701	51.0	Foci > 10	1710	36.7
	5294	100		4844	100		5291	100		4665	100

Experiment 4

Control noIR	No cells	%	L31 noIR	No cells	%	Control 3Gy 3h	No cells	%	L31 3Gy 3h	No cells	%
Foci = 0	1307	25.9	Foci = 0	1567	30.6	Foci = 0	711	13.4	Foci = 0	1221	24.1
Foci = 1	692	13.7	Foci = 1	965	18.8	Foci = 1	448	8.4	Foci = 1	768	15.1
Foci = 2	428	8.5	Foci = 2	519	10.1	Foci = 2	331	6.2	Foci = 2	467	9.2
Foci = 3	342	6.8	Foci = 3	363	7.1	Foci = 3	206	3.9	Foci = 3	266	5.2
Foci = 4	262	5.2	Foci = 4	277	5.4	Foci = 4	175	3.3	Foci = 4	217	4.3
Foci 5-9	1116	22.1	Foci 5-9	853	16.6	Foci 5-9	687	12.9	Foci 5-9	724	14.3
Foci > 10	903	17.9	Foci > 10	582	11.4	Foci > 10	2755	51.9	Foci > 10	1410	27.8
	5050	100		5126	100		5313	100		5073	100

Experiment 5

Control noIR	No cells	%	L31 noIR	No cells	%	Control 3Gy 3h	No cells	%	L31 3Gy 3h	No cells	%
Foci = 0	1765	37.6	Foci = 0	2239	47.1	Foci = 0	1291	28.3	Foci = 0	2106	44.2
Foci = 1	616	13.1	Foci = 1	1003	21.1	Foci = 1	504	11.1	Foci = 1	945	19.8
Foci = 2	330	7.0	Foci = 2	440	9.2	Foci = 2	238	5.2	Foci = 2	410	8.6
Foci = 3	228	4.9	Foci = 3	243	5.1	Foci = 3	175	3.8	Foci = 3	235	4.9
Foci = 4	218	4.6	Foci = 4	153	3.2	Foci = 4	132	2.9	Foci = 4	147	3.1
Foci 5-9	870	18.5	Foci 5-9	421	8.8	Foci 5-9	633	13.9	Foci 5-9	401	8.4
Foci > 10	666	14.2	Foci > 10	259	5.4	Foci > 10	1588	34.8	Foci > 10	524	11.0
	4693	100		4758	100		4561	100		4768	100

B

Control noIR

Control 3Gy 3h

L31 noIR

L31 3Gy 3h

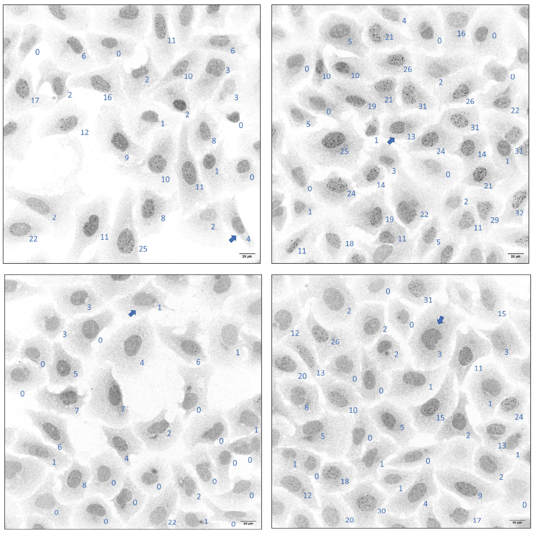

C

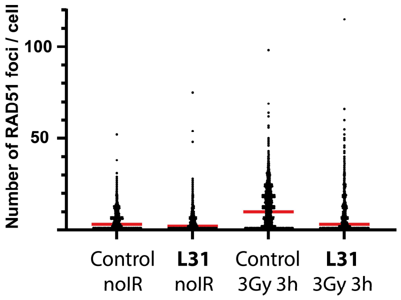

Figure S6. (**A**) Quantification of RAD51 foci in U2OS cells from five independent experiments in the presence of 40 µM **L31** or vehicle (control). (**B**) Representative IF images depicting RAD51 signal. Representative cells shown in **Figure S5A** are indicated with an arrow and they were chosen as the median RAD51 foci value for each of the images. Foci counts determined automatically using SImA software are provided next to each cell. Scale bar 20 µm. (**C**) Dot plot depicting the number of RAD51 foci per cell for one of the independent biological experiments. Red bars represent the mean values.

Mass spectra

Mass spectra of reaction optimisation conditions

Reaction **a**

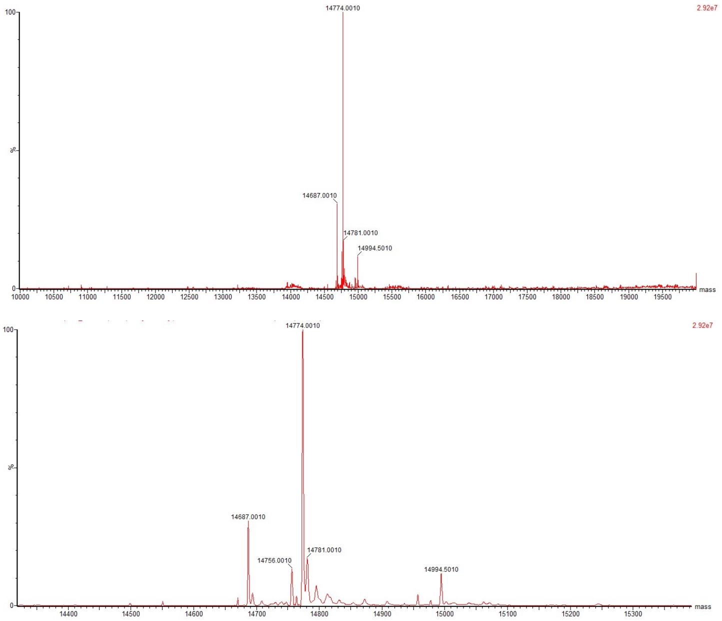

Figure S7. MS analysis of the reaction products of reaction **a**, for which the conditions are described in **Figure S2**

Reaction **b**

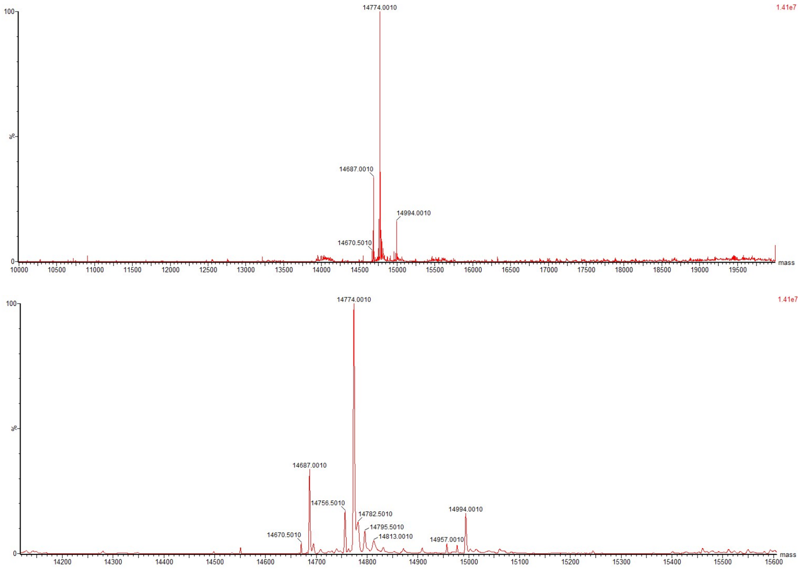

Figure S8. MS analysis of the reaction products of reaction **b**, for which the conditions are described in **Figure S2**

Reaction **c**

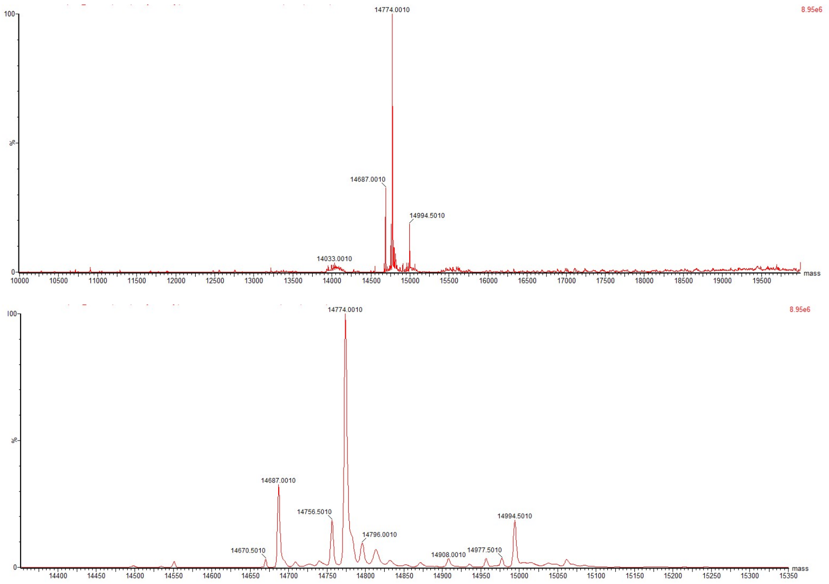

Figure S9. MS analysis of the reaction products of reaction **c**, for which the conditions are described in **Figure S2**

Reaction **d**

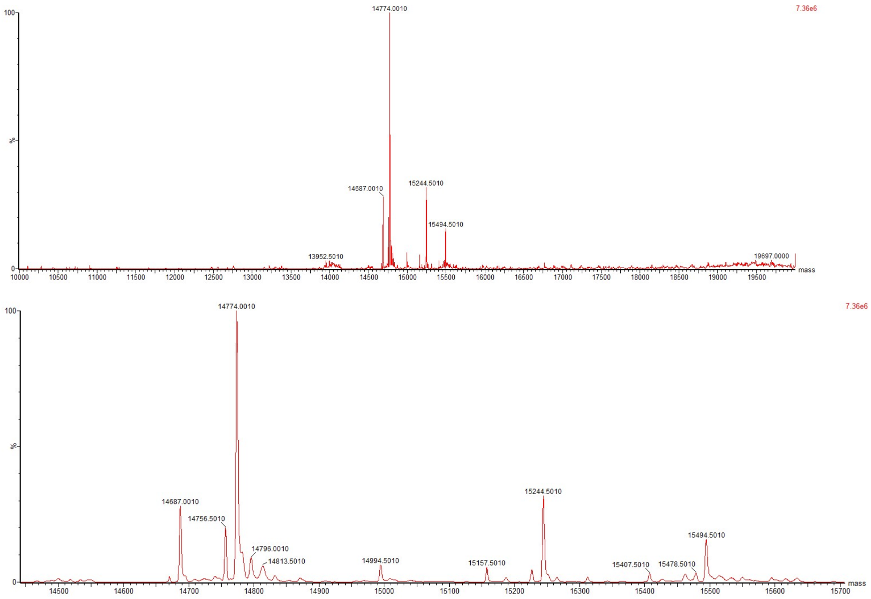

Figure S10. MS analysis of the reaction products of reaction **d**, for which the conditions are described in **Figure S2**

Reaction **e**

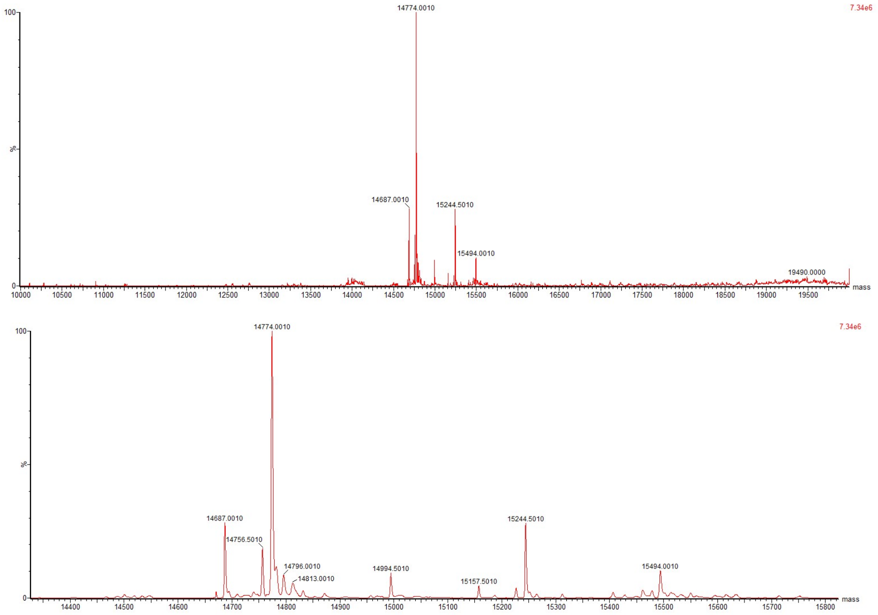

Figure S11. MS analysis of the reaction products of reaction **e**, for which the conditions are described in **Figure S2**

Reaction **f**

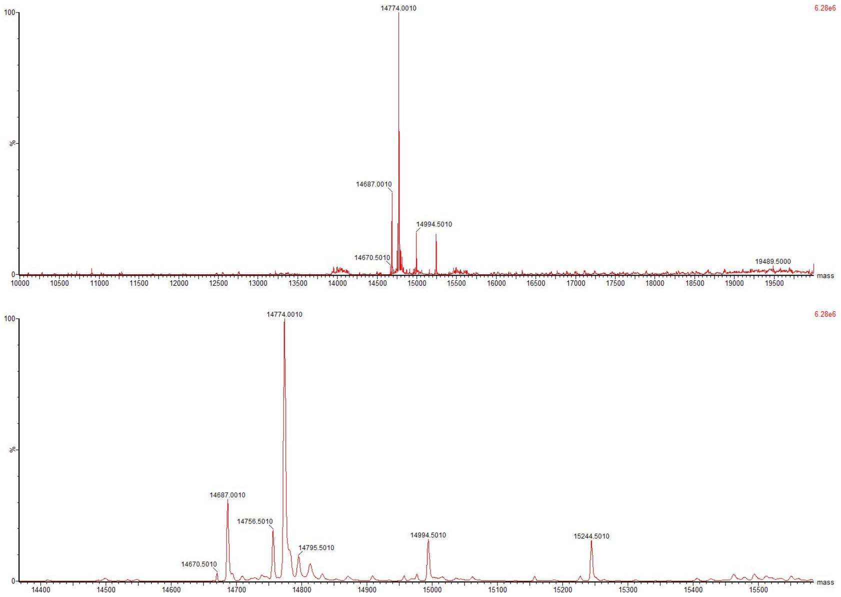

Figure S12. MS analysis of the reaction products of reaction **f**, for which the conditions are described in **Figure S2**

Reaction **g**

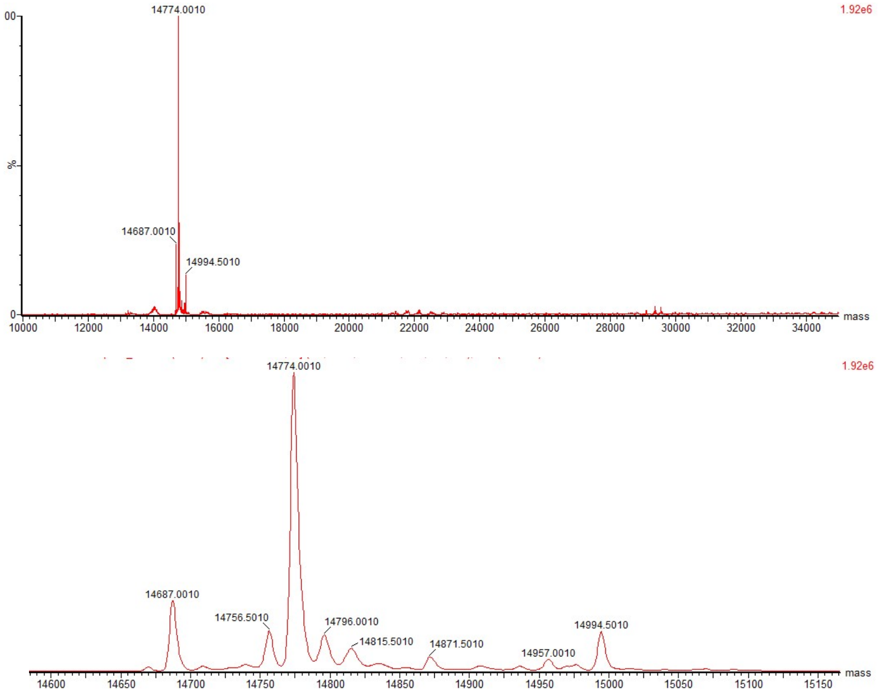

Figure S13. MS analysis of the reaction products of reaction **g**, for which the conditions are described in **Figure S2**

Reaction **h**

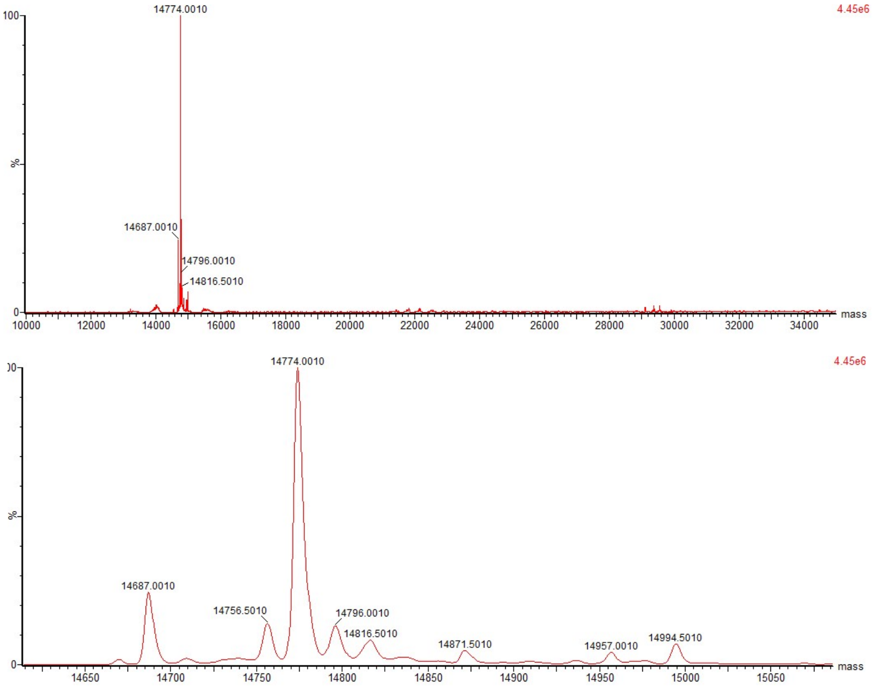

Figure S14. MS analysis of the reaction products of reaction **h**, for which the conditions are described in **Figure S2**

Mass spectra of representative GB1-fused stapled peptides

SP10

Species	mass (calc), Da	mass (obs), Da
GB1-**SP10**-DVT (-Met1)	14251.46	14251.5
GB1-**SP10**-DVT (-Met1, -Ser2)	14164.38	14164.5
GB1-**SP10**-$(DVT)_2$ (-Met1)	14471.69	14471.5

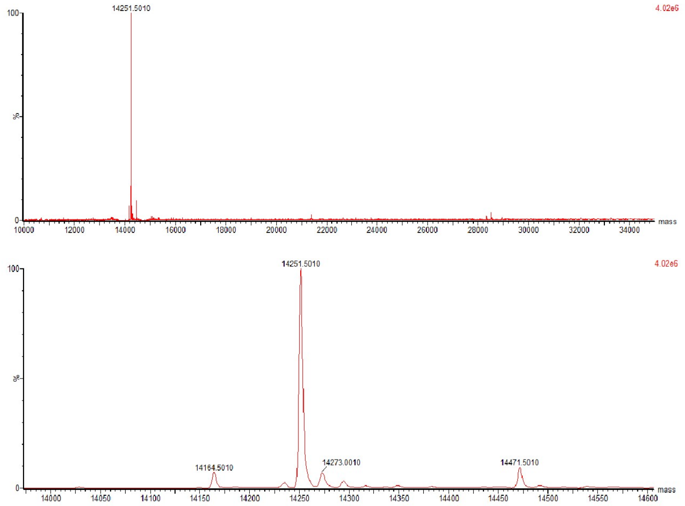

Figure S15. MS analysis of the GB1-fused peptide **SP10**.

SP11

Species	mass (calc), Da	mass (obs), Da
GB1-**SP11**-DVT (-Met1)	14210.4	14210.2
GB1-**SP11**-DVT (-Met1, -Ser2)	14123.33	14122.9
GB1-**SP11**-$(DVT)_2$ (-Met1)	14430.63	14430.4

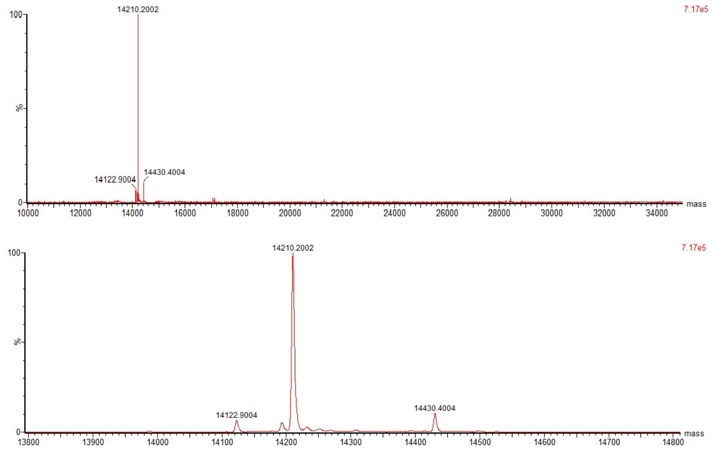

Figure S16. MS analysis of the GB1-fused peptide **SP11**.

SP12

Species	mass (calc), Da	mass (obs), Da
GB1-**SP12**-DVT (-Met1)	14500.64	14500.5
GB1-**SP12**-DVT (-Met1, -Ser2)	14720.87	14720.5
GB1-**SP12**-(DVT)$_2$ (-Met1)	14413.56	14413.5

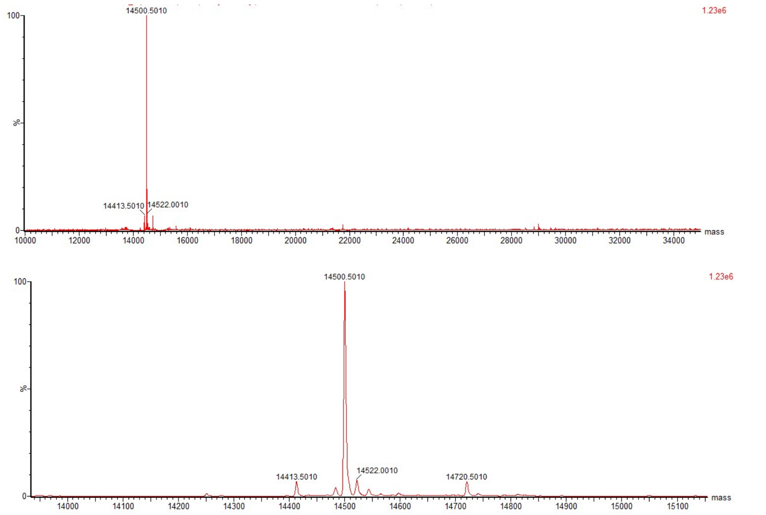

Figure S17. MS analysis of the GB1-fused peptide **SP12.**

SP15

Species	mass (calc), Da	mass (obs), Da
GB1-**SP15**-DVT (-Met1)	14760.97	14760.7
GB1-**SP15**-(DVT)$_2$ (-Met1)	14981.2	14981.2

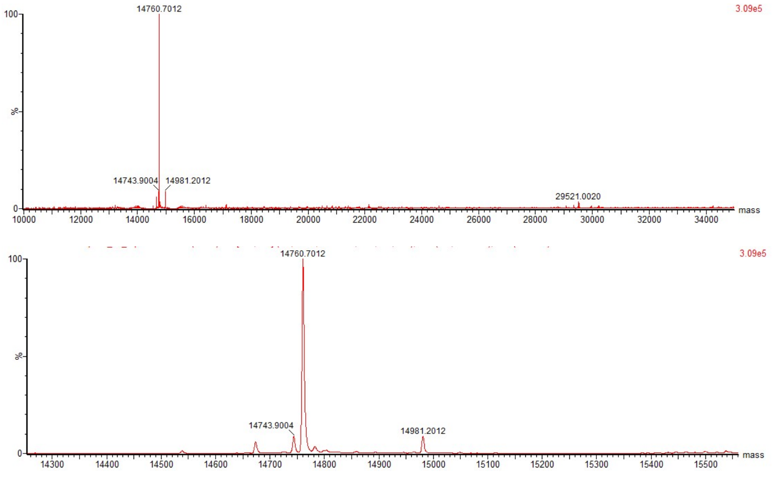

Figure S18. MS analysis of the GB1-fused peptide **SP15.**

Mass spectra of free stapled peptides

SP2

Species	m/z calculated	m/z found
M+2H	2289.97	2290.7
M+3H	1526.98	1527.5
M+4H	1145.48	1146.0
M+5H	916.59	917.0

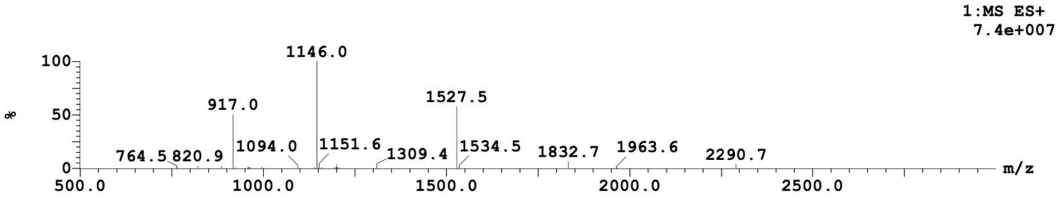

Figure S19. MS analysis of purified stapled peptide **SP2**.

SP24

Species	m/z calculated	m/z found
M+2H	1483.72	1483.3
M+3H	989.48	989.3
M+4H	742.36	742.0
M+5H	594.095276	594.0

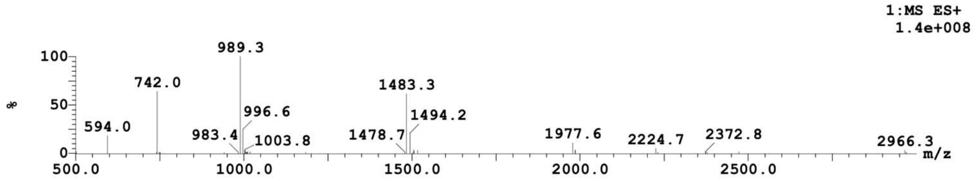

Figure S20. MS analysis of purified stapled peptide **SP24.**

SP30

Species	m/z calculated	m/z found
M+2H	1527.267276	1526.9
M+3H	1018.513943	1018.2
M+4H	764.137276	764.0
M+5H	611.511276	611.5

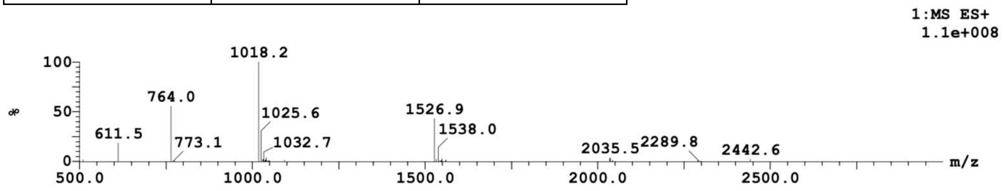

Figure S21. MS analysis of purified stapled peptide **SP30**.

SP31

Species	m/z calculated	m/z found
M+4H	1144.08	1144.0
M+5H	915.46	915.3
M+6H	764.58	762.9
M+7H	654.84	654.1

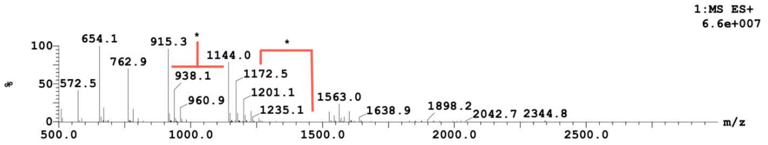

* = TFA counterions

Figure S22. MS analysis of purified stapled peptide **SP31**.

HPLC analysis of free peptides

SP2

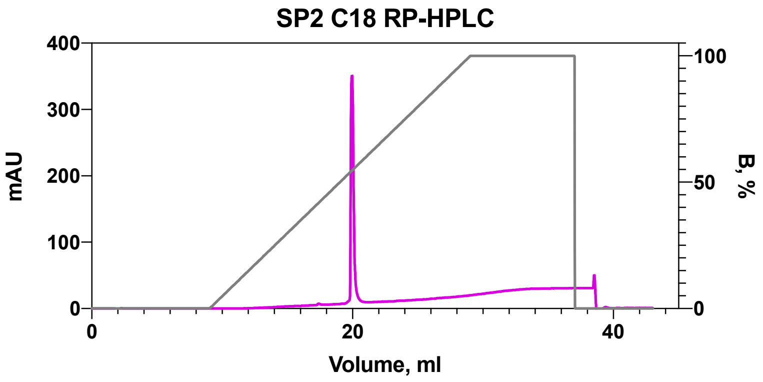

Figure S23. HPLC chromatogram of purified stapled peptide SP2.

SP24

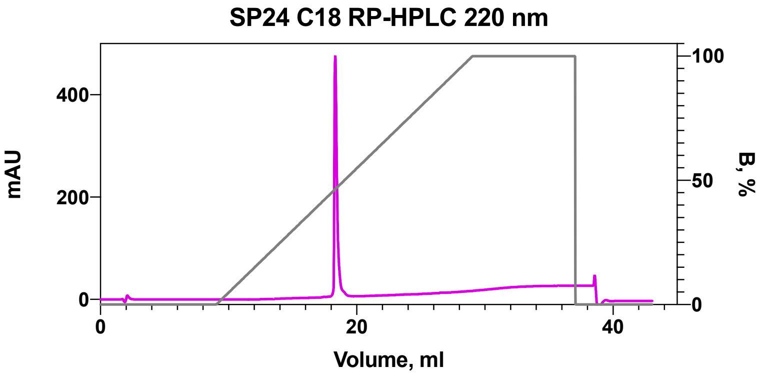

Figure S24. HPLC chromatogram of purified stapled peptide **SP24**.

SP30

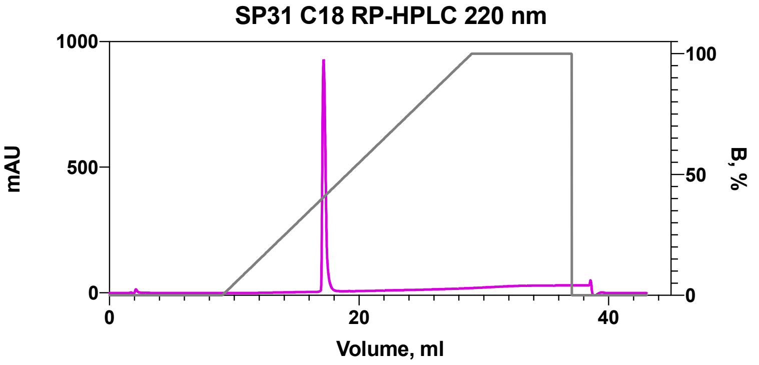

Figure S25. HPLC chromatogram of purified stapled peptide **SP30**.

SP31

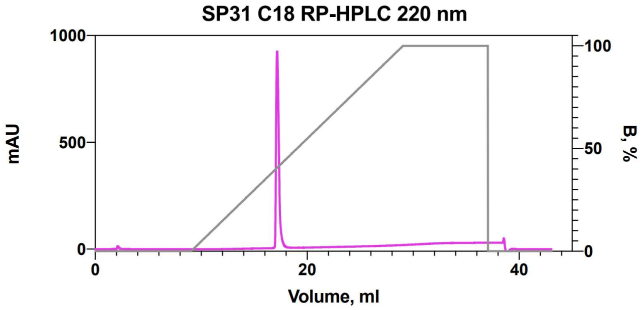

Figure S26. HPLC chromatogram of purified stapled peptide **SP31**.

References

[1] L. H. Lindenburg, T. Pantelejevs, F. Gielen, P. Zuazua-Villar, M. Butz, E. Rees, C. F. Kaminski, J. A. Downs, M. Hyvönen, F. Hollfelder, *Proc Natl Acad Sci USA* **2021**, *118*, e2017708118.

[2] T. Moschetti, T. Sharpe, G. Fischer, M. E. Marsh, H. K. Ng, M. Morgan, D. E. Scott, T. L. Blundell, A. R. Venkitaraman, J. Skidmore, C. Abell, M. Hyvönen, *Journal of Molecular Biology* **2016**, *428*, 4589–4607.

[3] S. J. Walsh, S. Omarjee, W. R. J. D. Galloway, T. T.-L. Kwan, H. F. Sore, J. S. Parker, M. Hyvönen, J. S. Carroll, D. R. Spring, *Chem. Sci.* **2019**, *10*, 694–700.

[4] N. S. Robertson, S. J. Walsh, E. Fowler, M. Yoshida, S. M. Rowe, Y. Wu, H. F. Sore, J. S. Parker, D. R. Spring, *Chem. Commun.* **2019**, *55*, 9499–9502.

[5] D. M. Hoover, *Nucleic Acids Research* **2002**, *30*, 43e–443.

[6] Z. Nikolovska-Coleska, R. Wang, X. Fang, H. Pan, Y. Tomita, P. Li, P. P. Roller, K. Krajewski, N. G. Saito, J. A. Stuckey, S. Wang, *Analytical Biochemistry* **2004**, *332*, 261–273.

[7] D. Liebschner, P. V. Afonine, M. L. Baker, G. Bunkóczi, V. B. Chen, T. I. Croll, B. Hintze, L.-W. Hung, S. Jain, A. J. McCoy, N. W. Moriarty, R. D. Oeffner, B. K. Poon, M. G. Prisant, R. J. Read, J. S. Richardson, D. C. Richardson, M. D. Sammito, O. V. Sobolev, D. H. Stockwell, T. C. Terwilliger, A. G. Urzhumtsev, L. L. Videau, C. J. Williams, P. D. Adams, *Acta Crystallogr D Struct Biol* **2019**, *75*, 861–877.

[8] P. Emsley, B. Lohkamp, W. G. Scott, K. Cowtan, *Acta Crystallogr D Biol Crystallogr* **2010**, *66*, 486–501.

[9] O. S. Smart, T. O. Womack, C. Flensburg, P. Keller, W. Paciorek, A. Sharff, C. Vonrhein, G. Bricogne, *Acta Crystallographica Section D* **2012**, *68*, 368–380.
